# Targeting the TREM1-positive myeloid microenvironment in glioblastoma

**DOI:** 10.1093/noajnl/vdac149

**Published:** 2022-09-15

**Authors:** Natalia Filippova, Jeffrey M Grimes, Jianmei W Leavenworth, David Namkoong, Xiuhua Yang, Peter H King, Michael Crowley, David K Crossman, L Burt Nabors

**Affiliations:** Department of Neurology, Division of Neuro-oncology, UAB, Birmingham, Alabama, USA; Department of Neurosurgery, Program of Immunology, UAB, Birmingham, Alabama, USA; Department of Neurosurgery, Program of Immunology, UAB, Birmingham, Alabama, USA; Department of Neurology, Division of Neuro-oncology, UAB, Birmingham, Alabama, USA; Department of Neurology, Division of Neuro-oncology, UAB, Birmingham, Alabama, USA; Department of Neurology, Birmingham Veterans Affairs Medical Center, Birmingham, Alabama, USA; Department of Genetics, Heflin Center Genomics Core, UAB, Birmingham, Alabama, USA (M.C., D.K.C.); Department of Genetics, Heflin Center Genomics Core, UAB, Birmingham, Alabama, USA (M.C., D.K.C.); Department of Neurology, Division of Neuro-oncology, UAB, Birmingham, Alabama, USA

**Keywords:** glioblastoma, heterogeneity, microenvironment, peri-necrotic

## Abstract

**Background:**

Tumor cellular and molecular heterogeneity is a hallmark of glioblastoma and underlies treatment resistance and recurrence. This manuscript investigated the myeloid-derived microenvironment as a driver of glioblastoma heterogeneity and provided a pharmacological pathway for its suppression.

**Methods:**

Transcriptomic signatures of glioblastoma infiltrated myeloid-derived cells were assessed using R2: genomic platform, Ivy Glioblastoma Spatial Atlas, and single-cell RNA-seq data of primary and recurrent glioblastomas. Myeloid-derived cell prints were evaluated in five PDX cell lines using RNA-seq data. Two immunocompetent mouse glioblastoma models were utilized to isolate and characterize tumor-infiltrated myeloid-derived cells and glioblastoma/host cell hybrids. The ability of an inhibitor of HuR dimerization SRI42127 to suppress TREM1^+^-microenvironment and glioblastoma/myeloid-derived cell interaction was assessed in vivo and in vitro.

**Results:**

TREM1^+^-microenvironment is enriched in glioblastoma peri-necrotic zones. TREM1 appearance is enhanced with tumor grade and associated with poor patient outcomes. We confirmed an expression of a variety of myeloid-derived cell markers, including TREM1, in PDX cell lines. In mouse glioblastoma models, we demonstrated a reduction in the TREM1^+^-microenvironment and glioblastoma/host cell fusion after treatment with SRI42127. In vitro assays confirmed inhibition of cell fusion events and reduction of myeloid-derived cell migration towards glioblastoma cells by SRI42127 and TREM1 decoy peptide (LP17) versus control treatments.

**Conclusions:**

TREM1^+^-myeloid-derived microenvironment promulgates glioblastoma heterogeneity and is a therapeutic target. Pharmacological inhibition of HuR dimerization leads to suppression of the TREM1^+^-myeloid-derived microenvironment and the neoplastic/non-neoplastic fusogenic cell network.

## Graphical Abstract

**Graphical Abstract:**
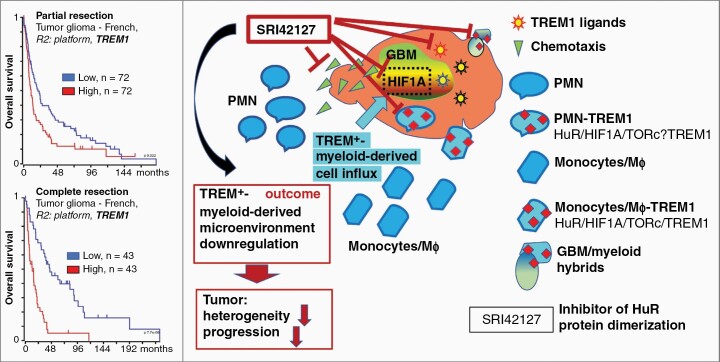

**Key Points**

- TREM1+-microenvironment is a hallmark of glioblastoma and tumor/myeloid-derived cell fusion.
- TREM1 is associated with infiltration of Mϕ and PMN cells in the peri-necrotic hypoxic zones.
- HuR dimerization inhibitor suppresses the TREM1+-microenvironment and tumor/host cell fusion.

**Importance of the Study**

The stress-response events, such as cell fusion and membrane nanotube formation that lead to intercellular gene-transfer and tumor plasticity, are poorly characterized in glioblastomas. Myeloid-derived immune-suppressive TREM1^+^-microenvironment promotes inflammatory stress, which positively correlates with disease progression. Our work highlights the role of the myeloid-derived TREM1^+^-glioblastoma microenvironment in the promulgation of hetero-cellular fusion events as an underlying mechanism of glioblastoma invasiveness and heterogeneity. We provide evidence that the mRNA-binding protein HuR could be one of the main regulators of the TREM1^+^-myeloid-derived axis in glioblastomas and that the pharmacological inhibition of HuR dimerization suppresses the TREM1^+^-glioblastoma microenvironment and cell fusion events in vivo and in vitro. This study advances the application of a recently discovered inhibitor of HuR dimerization for the suppression of oncogenic pro-inflammatory conditions associated with tumor development.

Circulating immune cells normally present at very low numbers in the CNS gain access to brain tissue affected by malignant transformations due to breach of blood–brain barrier (BBB) permeability and chemoattraction to the inflammatory glioma microenvironment.^1,2^ In a grade-dependent manner, pro-tumorigenic immune cells enrich the glioma microenvironment and often share genotype with tumor cells through intercellular gene-transfer.^2–4^ This contributes to de novo transformation, promoting tumor dissemination, generation of heterogeneity, reoccurrence, and treatment resistance.^4,5^

The focal hypoxic, ischemic, necrotic glioblastoma loci trigger a systemic inflammatory response and generate permissive niches that attract circulating immune cells.^1,2,6,7^ Among these cells are myeloid-derived peripheral immune cells that infiltrate the tumor microenvironment and selectively express an inflammatory type I membrane receptor (TREM1).^2,8^ TREM1 is activated by danger/damage-associated molecular patterns (DAMPs), like HMGB1, released from the brain into the systemic circulation and is up-regulated by the HuR-COX2-PGE2 axis provided by tumor cells locally.^9–13^ The cold-inducible RNA-binding protein (CIRBP), recently identified as a mediator of peritumoral invasion and a prognostic factor for recurrence of surgically resected brain metastases, is enriched in glioblastomas and is an endogenous TREM1 ligand.^14,15^ The extracellular actin (ACTB) and the peptidoglycan recognition protein 1 (PGLYRP1) are other TREM1 ligands associated with abnormal glioblastoma vascularity and neutrophil activation in damaged tissue.^8,10,16,17^ The sources of TREM1-positive cells reported in the glioblastoma microenvironment consist of monocytes and macrophages that emerged from monocytes recruited to the tumor bed, neutrophils, microglia, and endothelial cells.^2,8^ Indeed, we and others have shown improved patient outcomes with reduced neutrophils or neutrophil to lymphocyte ratios.^18–20^

Tumor cells with stemness hallmarks can fuse at a high rate with myeloid-derived cells in the inflammatory and hypoxic tumor microenvironment in different cancer types.^21–23^ A meta-analysis suggests that TREM1 up-regulation alters the expression of chemokines, cytokines, adhesive molecules, fusogens, and cytoskeletal remodeling transcripts that might, directly and indirectly, facilitate cell fusion events.^2,8,24^ The selective survival of the TREM1^+^-myeloid-derived cells is due to enhanced mitochondrial function promoted by Bcl2 and mitofusin overexpression.^25,26^ These events promote the development of treatment resistance and favorable survival of fused cells acquiring the TREM1^+^-phenotype.

The palette of TREM1 inhibitors is very poor and is represented by inhibitory peptides, which exhibit disadvantages due to limited BBB permeability and short lifetime. The significant overlap of cell signaling pathway and transcripts controlled by the mRNA-binding protein HuR with transcripts essential for stress-responsive TREM1 activation, myeloid-derived cell directional migration, and fusion creates the unique opportunity to override the TREM1-dependent complex myeloid-derived glioblastoma microenvironment with a recently discovered BBB permeable HuR inhibitor.^11–13,27–31^ Our current work explores the oncogenic role of the TREM1^+^-myeloid-derived glioblastoma microenvironment and presents a novel pharmacological opportunity for its inhibition.

## Materials and Methods

### Human Samples

The glioma and control brain tissue samples were obtained from O’Neal Tissue Procurement Shared Facility Brain Biorepository (IRB-300002497); the protein lysates were isolated as it was described.^30^ The study involved only the secondary analysis of human biological tissue and specimens were provided without identifiable information by personnel without any role in this research study.

### Mouse Glioblastoma Models and Treatments

All animal studies were carried out in compliance with UAB IACUC and NIH policies as we described^31^; see details in Supplementary material.

### Myeloid-Derived Cell Subsets Isolation and Analysis

The myeloid-derived cells were isolated from mouse brains harboring or not harboring tumors as described^32^; see details in Supplementary material. For the in vitro experiments, the myeloid-derived cell subsets (neutrophils and Mϕ) were isolated from the peritoneal lavage fluid as described^24^; see details in Supplementary material.

### Trans-Well Migration Assay, In Vitro

The *trans*-well migration assay was performed using polycarbonate BD Falcon FluoroBlok 24-multiwell Insert system, 3 µm (BD Biosciences Discovery Labware). Tumor cells (75–100 × 10^3^ per well) were platted in the bottom chamber 24 h prior to the experiment. The differentiated neutrophil-like HL-60 cells and Mϕ RAW267.4 were labeled with CellBrite Orange (Biotium, CA) according to the manufacturer’s instruction prior to the experiment. The primary neutrophils-RFP^+^ and Mϕ-RFP^+^ were isolated from the peritoneal lavage fluid of transgenic C57Bl/6 mice with wide-spread expression of tdTomato (RFP^+^) prior to the experiment (see protocol for myeloid-derived cell isolation from the peritoneal lavage fluid). During the experiment, both the insert and bottom chambers were loaded with the corresponding conditioned media. The myeloid-derived cells were resuspended and pre-incubated for 20 min in the conditioned media before loading to the insert chamber (100–150 × 10^3^ per well). The EVOSFI (Life Technologies) imaging system was utilized for myeloid-derived cell imaging and analysis on the insert and on the bottom chamber. At the end of the experiment, the insert and bottom chambers were separated for data processing. All conditions used were at least in duplicate for each experiment. Assays were performed in the presence of CoCl_2_ 132 µM to mimic the hypoxic condition.

### Cell Fusion Assay, In Vitro

#### Cell fusion assay between myeloid-derived cells and tumor adherent cells.

*—*Tumor cells expressing EGFP were plated in the complete DMEM/F12 media in 96-well plates 24 h before experiment. The myeloid-derived cells were marked with CellBrite Orange according to the manufacturer’s instruction. The neutrophil-like differentiated HL-60 cells and Mϕ RAW267.4 were resuspended in the conditioned media and added to the corresponding wells pre-loaded with tumor cells for 24–48 h. At the end of the experiment, cells were detached by trypsinization from each well, washed, and analyzed in the 4X well-size format (24-well plates) using EVOSFI imaging system.

#### Cell fusion assay between myeloid-derived cells and tumor neurospheres.

—Tumor neurospheres expressing EGFP were formed and maintained as previously described in Neurobasal-A medium (Gibco, Carlsbad, CA) supplemented with B-27 supplement without vitamin A (Gibco), N2 supplement (Gibco), 2 mM l-Glutamine (Mediatech) 100 U/ml Penicillin/Streptomycin (Mediatech), the basal growth factors EGF 20 ng/ml and bFGF, 20 ng/ml (TermoFisher, Waltham, MA) in 96-well plates [black with clear bottom (Corning, NY) and white with clear bottom (Kennebunk, ME)].^31^ The neutrophil-like differentiated HL-60 and Mϕ RAW267.4 cells were marked with CellBrite Orange according to the manufacturer’s instruction. The assays were performed in 96-well plates, the myeloid-derived cells were added to the tumor neurospheres for 48–72 h, 3D images were obtained with EVOSFI imaging system, each treatment condition was at least triplicate. The single-cell level analysis of fused cells was performed in the following steps: neurosphere/myeloid-derived cell complexes were collected, dissociated with Accutase (ThermoFisher), washed, and analyzed in the 4X well-size format (24-well plates) using EVOSFI imaging system. Multi nuclei formations were confirmed by staining with CellCycle-405blue (Invitrogen). The formation of multinuclear cells (MNC), the double-positive unencapsulated fluorescence signals, and TREM1 expression were the most distinctive features of fused cells presented in our models. Assays were performed in the presence of CoCl_2_ 132 µM to mimic the hypoxic condition or in the hypoxia incubator with the following hypoxic mixture: 5% O_2_, 10% CO_2_, 85% N_2._

### Proliferation Assay, In Vitro

Tumor neurospheres expressing EGFP were formed and maintained as previously described in black 96-well plates (Corning).^31^ The myeloid-derived cells (neutrophil-like differentiated HL-60 and Mϕ RAW267.4) were added to neurospheres for 24 h in the presence of (i) SRI42127, 5 µM or with corresponding vehicles as the control, (ii) with LP17 (35 ng/ml) or with the scrambled peptide (35 ng/ml) as the control (cells were pre-incubated with LP17 and control peptides for 20 min prior to the addition to the tumor neurospheres). The tumor EGFP-reporter signal was read by using a SynrtgyH1 microplate reader, images were taken using EVOSFI imaging system. The HL-60 cells were differentiated to the neutrophil-like cells in the RPMI (1x) + GlutaMAX medium (Gibco) supplemented with 100 U/ml Penicillin/Streptomycin (Mediatech), 10% FBS, 1.3%DMSO for 6 days prior experiment. Assay was performed in the presence of CoCl_2_ 132 µM. All conditions were used at least in triplicate.

### Immunohistochemistry

The immunostaining of the cell culture and tissue was performed as previously described^31^; see details in Supplementary material.

### HuR Dimerization Split Luciferase Reporter Assay

The reporter HuR dimerization split luciferase assay was performed as previously described in normal and hypoxic conditions with the following hypoxic mixture: 5% O_2_, 10% CO_2_, 85% N_2_.^30^

### PDX RNA-seq Data Generation and Analysis

Illumina global RNA-sequencing data were generated as described.^12,31^ Briefly, five patient-derived PDX neurosphere cell lines of different molecular subtypes (proneural PDX1, PDX2; classic PDX3, PDX4; mesenchymal PDX5) have been treated with SRI42127, 3 µM for 12 h or with vehicles as the control. RNA was isolated by using TRIzol reagent (Invitrogen, Carlsbad, CA) and processed in the UAB Genomic and Sequencing Core facility. The results were available in the Gene Expression Omnibus (GEO) repository database (GSE158271). Data were normalized to the RSP9 value in each sample; the experiments were performed twice for each cell line.

### Transcriptome Analysis and Datasets

The grade-dependent mini-ontology analysis of gene sets positively correlated with TREM1 gene expression was performed using the Madhavan study, R2: platform. Gene sets positively correlated with TREM1, *r* > .5, *P* < .009 cutoffs were used for the enrichment (Enrichr) analysis. A total of 454 genes for grades I–II, 241 genes for grade III, 198 genes for grade IV were selected for the analysis of biological processes, molecular function, cellular components, and pathway hallmarks using the Enrichr platform. GEO (GSE131928), the single-cell RNA-seq data from adult and pediatric glioblastomas, was used for the analysis of the TREM1 expression in Mϕ, malignant cells, oligodendrocytes, and T cell subsets using Broad Institute of MIT and Harvard portal (https://singlecell.broadinstitute.org/single_cell). GEO (GSE163120) and raw sequencing reads of the human scRNA-seq from European Genome-phenome Archive (EGA) (EGAS00001004871) were used for TREM1 profiling in TAMs from primary and recurrent glioblastomas. The Chinese Glioma Genome Atlas (CGGA) was used to analyze survival for patients harboring gliomas with high and low TREM1 expression. NIH GDC Data portal was utilized to obtain TCGA-GBM and TCGA-LGG digital slide collection and analysis of neutrophil infiltration in glioma tissue. cBioPortal was utilized to analyze correlation between Buffa-Hypoxia score and TREM1 expression in low and high grades gliomas.

### Statistical Analysis

For comparison between the two groups, Student’s two-tailed *t*-test was used. For comparisons between three or more groups, one-way ANOVA with multiple comparisons was used; a *P*-value less than .05 was considered to be significant. Correlation analysis was performed by using Pearson’s correlation. The results are shown as the mean ± SD.

## Results

### TREM1^+^-Microenvironment is Enriched in Glioblastoma and is Associated with Poor Patient Outcomes

**Figure 1.**
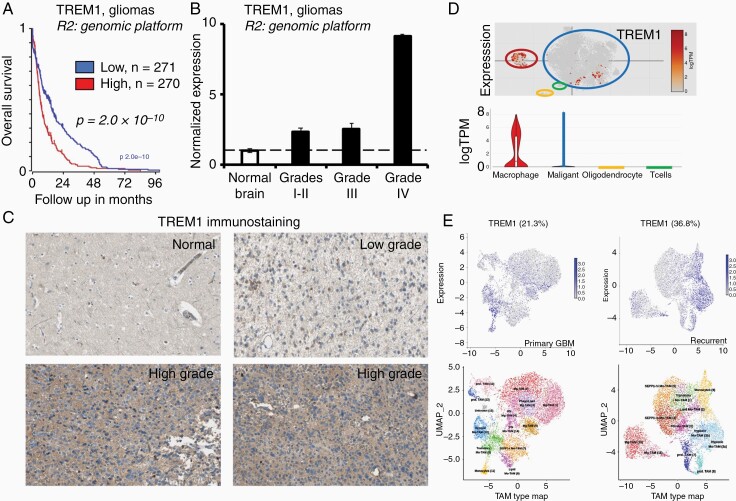
TREM1-expression in the glioblastoma microenvironment is associated with poor patient outcomes. (A) Kaplan–Meier overall survival curves (*x*-axis represents the time since glioma diagnostic) according to the tracks “high TREM1 expression, *n* = 270” versus “low TREM1 expression, *n* = 271” (median cut-off mode) are exhibited statistically significant differences (*P* = 2.0 × 10^−10^). (B) The graph illustrates an enhancement of the TREM1/ACTB mRNA ratios with the increase of the glioma grade. Results are shown as mean ± SD; the Student *t*-test determined significance; the difference is statistically significant between all glioma grades (*P* < .05). Data from the Madhavan study for brain tumors and the Harris study for normal brains were obtained from the R2: platform; normal brain (*n* = 44); grades I–II (*n* = 100); grade III (*n* = 85); grade IV (*n* = 143). (C) TREM1 immunostaining in normal and glioma tissues (HPA005563 anti-TREM1 antibody, MiliporeSigma, Human Protein Atlas database). (D) Single-cell TREM1 RNA-seq data from adult and pediatric glioblastomas, GEO: GSE131928. (E) TREM1 expression in myeloid-derived macrophages from primary and recurrent glioblastomas (Immune glioblastoma Atlas database).

First, we analyzed the clinical outcome of patients with gliomas harboring low or high expression of TREM1 by using the R2: Genomic Analysis and Visualization Platform (http://r2.amc.nl) Madhavan—550 MAS.5.0-u133p2 study; low expression levels of TREM1 were associated with favorable outcome (Figure 1A). A significant grade-dependent over-expression of TREM1 was confirmed in both low and high grades gliomas compared to normal brain samples utilizing the Madhavan study for brain tumors and Harris study for normal brain (Figure 1B). Note a significant TREM1 enhancement in high-grade WHO IV gliomas compared to low grades WHO I–II and grade III gliomas; TREM1 expression was normalized to the corresponding ACTB expression in each analyzed sample. Data from the Chinese Glioma Genome Atlas (CGGA) confirmed the highest TREM1 expression level in grade IV gliomas and a significant shortening of overall survival for patients harboring grade IV gliomas with TREM1 overexpression (Supplementary Figure 1A). Analysis of the TREM1 immunostaining in glioblastomas and normal brain tissues obtained from the Human Protein Atlas (http://www.proteinatlas.org) confirmed TREM1 overexpression in the glioblastoma microenvironment, particularly in peri-necrotic zones (Figure 1C). We found the strong Pearson correlation (0.53, *P* < .005) between TREM1 expression and Buffa-Hypoxia score in grade IV gliomas (Supplementary Figure 1B). Next, we utilized the single-cell RNA-seq data from adult and pediatric glioblastomas presented in the single-cell portal of the Broad Institute of MIT and Harvard (GEO: GSE131928),^33^ and confirmed TREM1 expression in both infiltrated Mϕ and tumor cells that imply intercellular gene-transfer between infiltrated myeloid-derived and tumor cells in the glioblastoma microenvironment (Figure 1D). TREM1 expression in the tumor-associated Mϕ (TAMs) from primary and recurrent glioblastomas obtained from the Brain Immune glioblastoma Atlas portal confirms an increase in the TREM1-positive subsets of TAMs from 21.3% in the primary glioblastoma to 36.8% in the recurrent glioblastoma (Figure 1E).

Therefore, data analysis suggests that the TREM1^+^-microenvironment supports glioma progression and is enhanced with tumor grade and glioblastoma recurrence.

### TREM1^+^-Myeloid-Derived Cells are Accumulated in the Peri-Necrotic Loci and are Involved in Cell Fusion Events with Glioblastoma Cells

**Figure 2.**
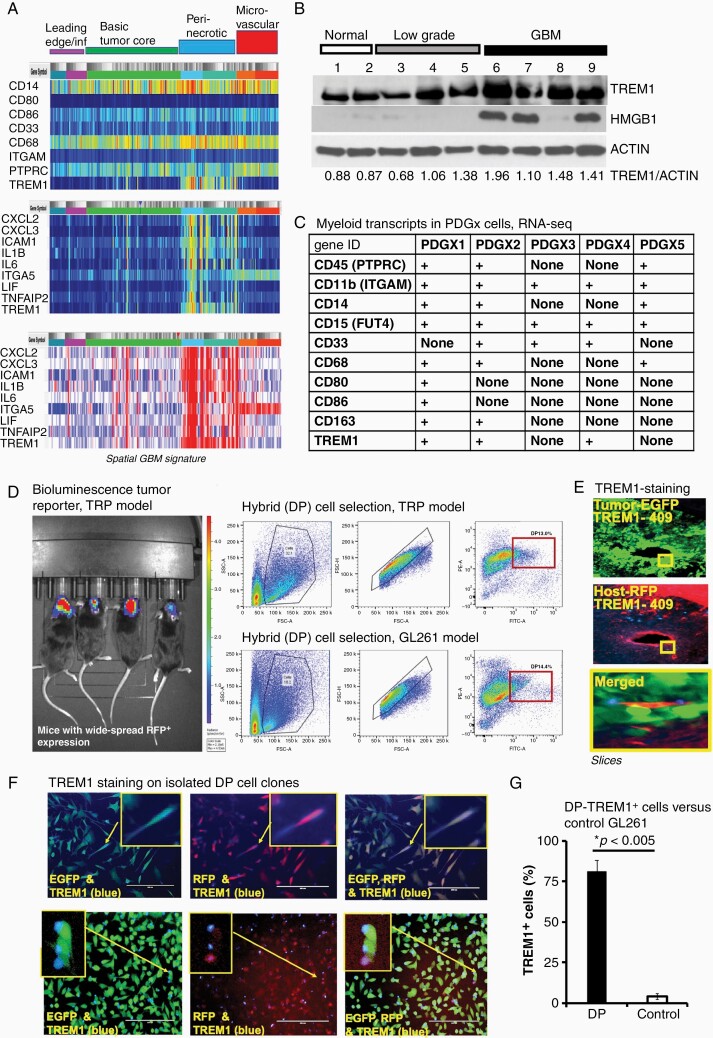
TREM1 expression is enriched in the glioblastoma peri-necrotic zones and in the glioblastoma/myeloid-derived cell hybrids. (A) Spatial RNA-seq signature of the myeloid-derived biomarkers and cytokines for glioblastomas of different molecular subtypes: classical, neural, proneural, mesenchymal, mesenchymal/neural, classical/neural, classical/mesenchymal, neural/proneural (36 samples total from 19 males and 17 females, Ivy Glioblastoma Project). The top two plots represent log2 of transcript expression; the bottom plot illustrates *z*-score. Note that the TREM1 is enriched in the peri-necrotic zones of glioblastomas of all molecular subtypes. (B) Western blot illustrates TREM1 and HBGB1 protein levels in normal and glioma samples of different grades; Actin is shown to confirm equal protein loading, TREM1/Actin ratios were calculated by using Image J program and are shown for each sample. (C) Chart illustrates expression of the myeloid cell markers in PDX cell lines of different subtypes (classical PDX1, PDX2; neural PDX3, PDX4; and mesenchymal PDX5; RNA-seq data). (D) Representative plots illustrate DP (double-positive, EGFP^+^RFP^+^) cell selection by flow cytometry technique from dissociated tumor tissue from the immunocompetent glioblastoma mouse models; TRP-Fluc-EGFP and GL261-EGFP cells were intracranially injected in the immunocompetent mice with a wide-spread expression of RFP; DP cells were isolated 24 days after tumor implantation (see method). The representative image illustrates the bioluminescence tumor-reporter signal from mice injected with TRP-Fluc-EGFP cells. (E) Images illustrate representative TREM1 immunostaining in brain slices from glioblastoma mouse model with host cells expressing RFP and GL261-EGFP tumor; scale bar is 200 µm; the insert illustrates TREM1 expression on the host cell, which is undergoing fusion with tumor-cell in peri-necrotic zone. (F) Images illustrate representative TREM1 immunostaining in cultured DP (EGFP^+^RFP^+^) cells isolated from the immunocompetent glioblastoma model with a wide-spread expression of RFP in host cells and GL261-EGFP tumor. Multi nuclei formations were observed in around 90% of DP cells. The scale bar is 200 µm. (G) Graph illustrates the percentages of the TREM1^+^-cells in DP (EGFP^+^RFP^+^) glioblastoma/host hybrids versus control GL261-EGFP cells. Results are shown as mean ± SD; the difference is statistically significant (*P* < .005, Student *t-*test). At least five visual fields were analyzed for each analyzed slice, or sample of DP cells per mouse.

We employed RNA-seq data from Ivy Glioblastoma Project (http://glioblastoma.alleninstitute.org) to analyze the spatial distribution of the myeloid-cell markers and chemokines in the glioblastoma microenvironment. An increase of TREM1 was confirmed in the peri-necrotic areas of all glioblastoma molecular subtypes (Figure 2A). In the same zone, we found an accumulation of the monocyte and macrophage markers as well as chemokines like CXCL2 and CXCL3 known to be involved in the recruitment of polymorphonuclear leukocytes (PMN). The appearances of PMN were confirmed in peri-necrotic and perivascular zones in GBMs (three or less per visual fields) and to a lower extent (one or less per visual fields) in lower-grade gliomas using TCGA-GBM and TCGA-LGG digital slide collection. An increase in TNFAIP2, ICAM1, and ITGA5 expression pointed to a possible TREM1- and HuR-dependent formation of tertiary structures between tumor and myeloid-derived cells within the inflammatory peri-necrotic areas. A grade-dependent enhancement of the TREM1/HMGB1 axis in the glioblastoma microenvironment compared to normal brains was confirmed using clinical tissue samples and western blot technique (Figure 2B). Note that the HMGB1 protein belongs to the DAMPs released by transformed cells and is involved in the myeloid-derived cell chemoattraction and TREM1-activation.^9^ Next, we performed profiling of the human myeloid-derived cell markers in patient-derived glioblastoma xenolines (PDX) using RNA-seq technique. The RNA-seq data analysis of five PDX cell lines of different molecular subtypes (classic, mesenchymal, and proneural) revealed expression of a variety of myeloid-cell markers (including CD45, CD11b, CD14, CD15, CD33, CD163, CD68, CD80, CD86) following at least five passages of PDX cell lines in the cell culture (Figure 2C). This supports the hypothesis of intercellular gene-transfer between glioblastoma and myeloid-derived cells in the glioblastoma microenvironment. TREM1 expression was detected in three of five PDX cell lines and in the established U87-MG glioblastoma cell line.

To evaluate cell fusion events in the glioblastoma microenvironment, we employed two immunocompetent glioblastoma models with fluorescently defined host and glioblastoma cells. The established mouse glioblastoma GL261-EGFP cell line and mouse TRP-EGFP-Fluc cell line of transformed astrocytes (truncated SV40 T antigen, Kras G12D mutation, Pten deletion)^32^ were intracranially injected in the transgenic C57Bl/6 mice with wide-spread expression of tdTomato (RFP^+^). Figure 2D illustrates the gating of the double-positive (DP) glioblastoma/host hybrid cells (EGFP^+^RFP^+^), isolated from the dissociated brain tissues using a 30% Percoll gradient. The DP cell percentage varied from 1 to 14% and positively correlated with tumor size. TREM1 expression on host cells undergoing glioblastoma/host cell fusion was confirmed on brain slices prepared from the GL261 glioblastoma mouse model (Figure 2E). Around 75% of DP cells, expanded in cell culture and stained with the TREM1 antibody, were TREM1 positive compared to less than 1% of control cells (Figure 2F, G). The multinuclear formations, the DP unencapsulated fluorescence signals, and TREM1 expression were the most distinctive features of fused cells in our model.

Therefore, our results confirm involvement of the myeloid-derived microenvironment in cell fusion and spatial accumulation of TREM1^+^-cells in the peri-necrotic glioblastoma loci.

### Suppression of the TREM1^+^-Myeloid-Derived Microenvironment and Cell Fusion Events in the Glioblastoma Mouse Models by an Inhibitor of HuR Dimerization

**Figure 3.**
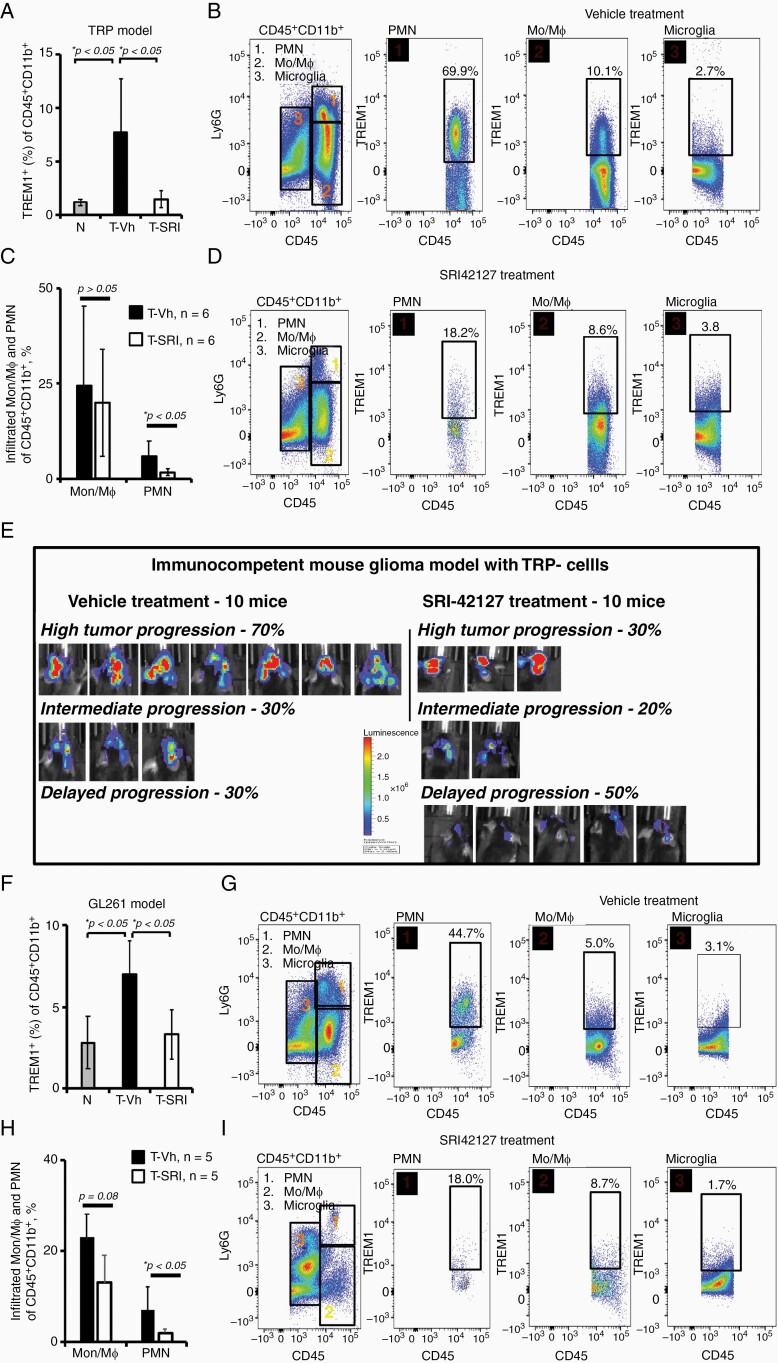
TREM1^+^-myeloid-derived glioblastoma microenvironment is downregulated by an inhibitor of HuR dimerization in immunocompetent glioblastoma mouse models. (A, F) Graphs illustrate the weights of TREM1^+^-myeloid-derived microenvironment in the dissociated brain tissue from immunocompetent TRP and GL261 mouse glioblastoma models at different treatment modalities versus normal (not tumor harboring) mice. Results are shown as mean ± SD; the statistically significant differences are marked with asterisks (*P* < .05, Student *t-*test). The percentages of TREM1^+^-myeloid-derived cells were the following in the experiment with TRP glioblastoma model: 1.2 ± 0.3%, *n* = 3 in normal mice without intracranial tumor injection, 7.7 ± 5.0%, *n* = 6 in mice injected with TRP tumor cells and treated with control vehicles, 1.5 ± 0.8%, *n* = 6 in mice intracranially injected with TRP tumor cells and treated with SRI42127. The percentages of TREM1^+^-myeloid-derived cells were the following in the experiment with GL261 glioblastoma model: 2.8 ± 1.6%, *n* = 3 in normal mice without intracranial tumor injection, 7.0 ± 1.2%, *n* = 5 in mice intracranially injected with GL261 tumor cells and treated with control vehicles, 3.3 ± 1.5%, *n* = 5 in mice injected with GL261 cells and treated with SRI42127. (B, G) Representative plots illustrate flow cytometric profiling of brain myeloid-derived microenvironment from TRP and GL261 immunocompetent glioblastoma mouse models 20 days post intracranial tumor implantation. Plots illustrate representative examples of TREM1 expression in different subsets of myeloid-derived cells isolated from each group of mice (plots of each set represent data from the same mouse). (C, H) Graphs illustrate the weights of PMN and Mϕ cell subsets in brain tissue from TRP(C) and GL261(H) glioblastoma models at different treatment modalities. Results are shown as mean ± SD; the statistically significant differences are marked with asterisks (*P* < .05, Student *t-*test). The percentages of tumor-infiltrated PMN and Mϕ were the following in the TRP-glioblastoma model: 5.7 ± 4.4%, *n* = 6 and 24 ± 22%, *n* = 6 after control-vehicle treatment versus 1.5 ± 0.9%, *n* = 6 and 20 ± 14%, *n* = 6 after SRI42127 compound treatment. The percentages of tumor-infiltrated PMN and Mϕ were the following in the GL261-glioblastoma model: 23 ± 9%, *n* = 5 and 7 ± 3%, *n* = 5 after control-vehicle treatment versus 13 ± 6, *n* = 5 and 1.9 ± 1, *n* = 5 after SRI42127 compound treatment. (D, I) Representative plots illustrate flow cytometric profiling of myeloid-derived tumor microenvironment in TRP and GL261 glioblastoma models treated with SRI42127 compound. Plots illustrate representative examples of TREM1 expression in different subsets of myeloid-derived cells isolated from each group of mice (plots inside of each set represent data from the same mouse). (E) Images of bioluminescence tumor-reporter signals from TRP glioblastoma model after treatment with SRI42127 compound (10 mice) versus control treatment with vehicles (10 mice) twenty days post intracranial tumor implantation. Note the robust tumor-reporter signal from 70% of mice in the control group versus 30% of mice in the group treated with SRI42127, suggesting the delay of the tumor progression in the mouse group treated with SRI42127.

First, we defined the TREM1^+^-myeloid-derived microenvironment in the immunocompetent TRP and GL261 mouse glioblastoma models. TREM1 expression was significantly enhanced in the myeloid-derived (CD45^+^CD11b^+^) cells isolated from the dissociated brain tissue of mice harboring TRP and GL261 tumors compared to the control mice without intracranial tumor cells injection (Figure 3A, F for TRP and GL261 glioblastoma models, respectively). TREM1^+^-cells were enriched in poly-morphonuclear PMN (up to 75%) and Mϕ (up to 15%) fractions, and at a lesser degree were observed in the microglia fraction (up to 3%) following three weeks of the tumor development (Figure 3B, G for TRP and GL261 glioblastoma models, respectively). Supplementary Figure 2 illustrates the main characteristics of tumor-infiltrated myeloid-derived cells exhibiting TREM1 expression. In summary, M2 CD206^+^-subsets of Mϕ mostly expressed TREM1 in both GL261 and TRP glioblastoma models and were immunosuppressive, positive for PD-L1 (see details in the Supplementary Figure 2A, B); GL261 model revealed the unique subset of PMN with strong positive correlation between TREM1 and PDL1 expression (*R* > .5, *P* < .005), TREM1^+^PDL1^+^ subsets reached up to 35% and 50% of PMNs for GL261 and TRP models, respectively (Supplementary Figure 2C). Recently, we developed a new inhibitor of the mRNA-binding protein HuR dimerization SRI42127 and confirmed that HuR dimerization is involved in generation of the myeloid-derived microenvironment following LPS-induced pro-inflammatory conditions.^27,31^ We predicted that the pro-inflammatory myeloid-derived microenvironment could be affected by the SRI42127 compound in glioblastoma and therefore, evaluated the composition of the myeloid-derived microenvironment in the immunocompetent TRP and GL261 glioblastoma mouse models treated with SRI42127 compound (15 mg/kg, twice per day for 20 days) versus the control (vehicle treatment). We confirmed a significant decrease in the TREM1^+^-myeloid-derived microenvironment after SRI42127 treatment in both models (Figure 3A, D, F, I for TRP and GL261 glioblastoma models, respectively); TREM1 decline was accompanied by a significant reduction in tumor-infiltrated PMN cells and notable but not significant reduction of Mϕ (Fig. 3C, H for TRP and GL261 glioblastoma models, respectively). SRI42127 significantly reduced the population of TREM1^+^PDL1^+^ subpopulation of PMN cells, which exhibited a strong positive correlation between TREM1 and PDL1 expression, from 5.5% ± 1% (*n* = 5) to 2.1% ± 0.9% (*n* = 5), *P* < .005 of total CD45^+^CD11b^+^cells (Supplementary Figure 2C). Overall, the PDL1^+^-immunosuppressive myeloid-derived microenvironment exhibited a tendency to reduction after SRI42127 treatment (see details in Supplementary Figure 2C–E).

Following 20 days post intracranial TRP-cells implantation, mice treated with control vehicles exhibited spatial and robust tumor-reporter bioluminescence signals in 70% of mice (Figure 3E); these tumors were defined as high-grade tumors with pathology closely resembling high-grade gliomas in human patients.^34^ Mice treated with SRI42127compound exhibited high-grade tumors only in 30% of mice and exhibited delayed tumor progression characterized by limited tumor-reporter bioluminescence signals and smooth/non-infiltrating tumor edges in 50% of mice (Figure 3E). In the glioblastoma GL261 model, we observed the reductions in tumor invasion in the mouse group treated with the SRI42127 compound versus the control mouse group treated with vehicles (Supplementary Figure 3). Therefore, our results confirm that the TREM1^+^-myeloid-derived microenvironment in the glioblastoma mouse models is mostly represented by PMN and Mϕ; the inhibitor of the HuR dimerization might suppress it.

**Figure 4.**
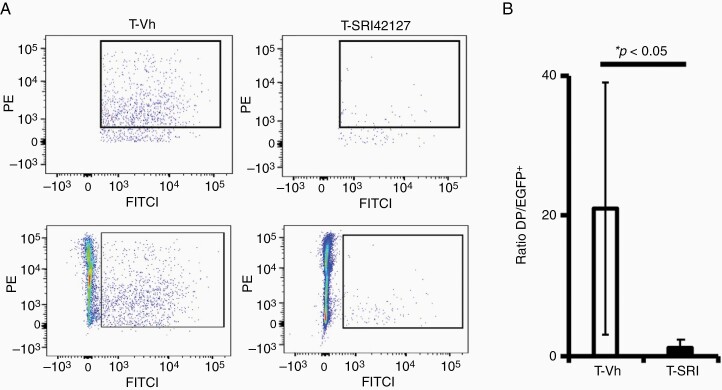
Glioblastoma/host cell fusion events are significantly reduced by SRI42127 in the immunocompetent GL261 glioblastoma model. (A) Representative plots (top) of DP (EGFP^+^RFP^+^) glioblastoma/host cell hybrids selected by flow cytometry technique from brain tissue of GL261 mouse glioblastoma model treated with SRI42127 (T-SRI42127) versus control-vehicle treatment (T-Vh). Bottom plots illustrate corresponding EGFP^+^RFP^+/−^ cell selection. GL261-EGFP cells (0.8 × 10^6^) were injected in ten mice with wide-spread expression of RFP; mice were randomly divided into two equal groups and treated for 18 days starting from day four with SRI42127 (15 mg/kg, twice per day) or control vehicle. (B) The graph represents (EGFP^+^RFP^+^)/(EGFP^+^RFP^−^) cell ratio in the mouse group treated with SRI42127 versus the control group. Results are shown as mean ± SD; the difference is statistically significant (*P* < .05, Student *t-*test).

To determine whether the suppression of the TREM1^+^-glioblastoma microenvironment leads to a reduction in cell fusion events, we analyzed cell fusion events between tumor and myeloid-derived cells in the fluorescently defined GL261 glioblastoma model following mice treatment with SRI42127 (15 mg/kg, twice per day for 18 days) versus control treatment. The immunocompetent C57Bl/6 mice with wide-spread expression of tdTomato (RFP^+^) were intracranially injected with GL261-EGFP cells, and the ratios of DP (EGFP^+^RFP^+^) cell hybrids isolated by using 30% Percoll gradient to unfused tumor cells (EGFP^+^RFP^−^) were determined using flow cytometry. Figure 4A illustrates representative examples of DP cell gating in the SRI42127 and control mouse groups; note a significant reduction in the (EGFP^+^RFP^+^)/(EGFP^+^RFP^−^) cell ratio in the SRI42127 group versus control (Figure 4B).

Thus, prevention of HuR dimerization leads to downregulation of the TREM1^+^-glioblastoma microenvironment and is associated with a reduction in cell fusion events between tumor and myeloid-derived cells in the mouse glioblastoma model.

Chemotaxis of neutrophils and Mϕ towards glioma cells is attenuated by the inhibitor of HuR dimerization SRI42127 and the TREM1-decoy peptide LP17 in vitro.

**Figure 5.**
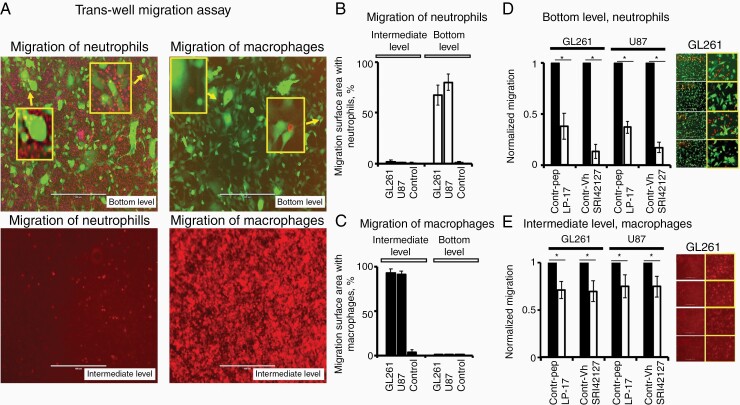
Chemotaxis of myeloid-derived cells towards glioblastoma cells was attenuated by HuR and TREM1 inhibitors at the mimic hypoxic condition in vitro. (A) Representative images of migration of neutrophils and Mϕ labeled with red-fluorescence dye towards glioblastoma GL261-EGFP cells in the trans-well migration assay. Scale bars are 400 um in all images. (B, C) The graphs represent the areas (in percentages) covered by migrated neutrophils and Mϕ on the middle filter and at the bottom level in the trans-well migration assays towards glioblastoma GL261 and U87 cells plated at the bottom level 24 h prior to neutrophil and Mϕ placement in the middle chamber. Control experiments were performed without tumor cells on the bottom level. Results are shown as mean ± SD. (D, E) Graphs represent normalized migration of neutrophils and Mϕ towards glioblastoma cells in trans-well migration assay at different treatment modalities. The normalized values of neutrophil migration (detected on the bottom level) were 0.38 ± 0.13, *n* = 7 and 0.37 ± 0.06, *n* = 4 in the presence of LP17 (35 ng/ml) peptide towards GL261 and U87 cells, respectively, versus control-peptide treatments; and 0.13 ± 0.07, *n* = 7 and 0.17 ± 0.05, *n* = 7 in the presence of SRI42127 compound (5 µM) towards GL261 and U87 cells, respectively, versus control-vehicle treatments. The differences were significant for all conditions (*P* < .05, Student *t-*test). The normalized values of Mϕ migration (detected in the middle filter) were 0.71 ± 0.09, *n* = 7 and 0.75 ± 0.12, *n* = 4 in the presence of LP17 (35 ng/ml) peptide towards GL261 and U87 cells, respectively, versus control-peptide treatments; and 0.70 ± 0.11, *n* = 7 and 0.75 ± 0.11, *n* = 5 in the presence of SRI42127 (5 µM) towards GL261 and U87 cells, respectively, versus control-vehicle treatments. The differences were significant for all conditions (*P* < .05, Student *t*-test). The images on the right side of the graphs illustrate representative examples of myeloid-derived cell migration under different treatment conditions; scale bars are 400 µm; inserts framed with yellow represent images at high magnification.

We used established myeloid-derived cell line models of neutrophils (differentiated HL-60 cells)^35^ and MϕRAW264.7 to evaluate the impact of TREM1-decoy peptide LP17 and the HuR dimerization inhibitor SRI42127 on cell chemotaxis towards glioblastoma cells in the trans-well assay under conditions that mimic hypoxia (CoCl_2_ 132 uM) in vitro (Figure 5A–E). Figure 5A illustrates the migration of neutrophils and Mϕ, labeled with the red-fluorescent dye, towards GL261-EGFP cells in the trans-well migration assay. The neutrophil migration was evaluated 12 h after cell placement into the middle chamber; the Mϕmigration was evaluated after 24–48 h of cell placement into the middle chamber. Note that neutrophils migrated through the pores of the insert and reached the bottom chamber (bottom level), loaded with tumor cells. Mϕmigrated through the pores of the insert and remained on the other side of the insert surface (intermediate level); less than 0.00001% of macrophages reached the bottom level. Neither neutrophils nor Mϕexhibited directional migration in the control condition (without tumor cells). Graphs in Figure 5B and C represent average data of neutrophil and Mϕmigration towards GL261 and U87 cell lines. A significant reduction in directional migration of neutrophils and to lesser degree of Mϕ was observed in the presence of SRI42127 compound (5 µM) and LP17-peptide (35 ng/ml) compared to the vehicle and scrambled control-peptide (35 ng/ml) treatments, respectively (Figure 5D, E). Representative images of neutrophil and Mϕ migrations towards GL261 cells at different treatment conditions are shown in Figure 5D and E on the right side of the graphs. Next, primary neutrophils and Mϕ isolated from the thioglycolate-stimulated peritoneal cavities of C57Bl/6 mice with wide-spread expression of tdTomato were evaluated in trans-well migration assay with Gl261 cells under conditions that mimic hypoxia. Primary neutrophils and Mϕexhibited a significant reduction in migration towards glioblastoma cells in the presence of LP17-peptide (35 ng/ml) and SRI42127 (5 µM) compared to the control-peptide (35 ng/ml) and vehicle treatments, respectively (Supplementary Figure 4).

Therefore, our data suggest that cell signaling pathways controlled by HuR dimerization and TREM1 activation are involved in myeloid-derived cell migration toward tumor cells in the hypoxic condition.

### Intercellular Cell Fusion is Attenuated by the Inhibitor of HuR Dimerization SRI42127 and the TREM1-Decoy Peptide LP17 In Vitro

**Figure 6.**
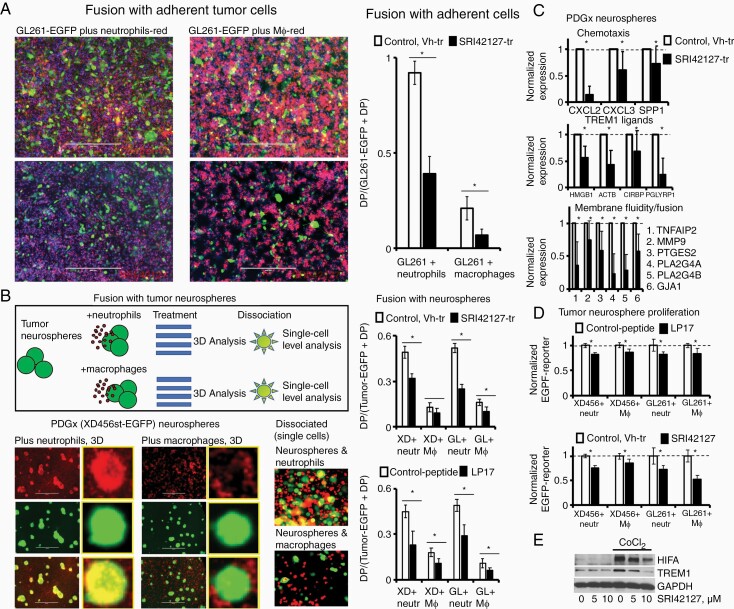
Cell fusion events between glioblastoma and myeloid-derived cells were significantly attenuated by HuR and TREM1 inhibitors at the mimic hypoxic condition in vitro. (A) Representative images of cell fusion events between myeloid-derived cells labeled with red-fluorescence dye and adherent glioblastoma GL261-EGFP cells at control (vehicle treatment) and SRI42127 (5 µM) treatment conditions. Scale bars are 400 µm. (B) Schematic illustration of cell fusion experiment between myeloid-derived cells labeled with red-fluorescence dye and tumor neurospheres expressing EGFP (top panel); representative images of cell fusion events between myeloid-derived cells labeled with red-fluorescence dye and PDX (XD456-EGFP) tumor neurospheres at 3D and single-cell levels (bottom panel). Scale bars are 400 µm. Yellow framed inserts represent images of neurospheres at high magnification. The graphs represent ratios of DP (EGFP^+^RFP^+^) to (EGFP^+^ + EGFP^+^RFP^+^) cells in cell fusion assays between myeloid-derived cells and tumor neurospheres at different treatment conditions: SRI42127 treatment versus control (vehicle treatment) and LP17 treatment versus control scrambled peptide treatment. The ratios were the following: 0.49 ± 0.04, *n* = 6 (Vh-tr), 0.32 ± 0.03, *n* = 6 (SRI42127-tr) and 0.13 ± 0.03, *n* = 4 (Vh-tr), 0.09 ± 0.03, *n* = 4 (SRI42127-tr) in experiments with XD456 neurospheres and neutrophils or Mϕ, respectively; 0.52 ± 0.03, *n* = 4 (Vh-tr), 0.25 ± 0.03, *n* = 4 (SRI42127-tr) and 0.16 ± 0.02 *n* = 4 (Vh-tr), 0.10 ± 0.03, *n* = 4 (SRI42127-tr) in experiments with GL261 neurospheres and neutrophils or Mϕ, respectively; 0.45 ± 0.04, *n* = 6 (control-peptide), 0.23 ± 0.09, *n* = 6 (LP17) and 0.18 ± 0.03, *n* = 6 (control-peptide), 0.11 ± 0.03, *n* = 6 (LP17) in experiments with XD456 neurospheres and neutrophils or Mϕ, respectively; 0.49 ± 0.04, *n* = 5 (control-peptide), 0.29 ± 0.07, *n* = 5 (LP17) and 0.11 ± 0.03, *n* = 5 (control-peptide), 0.06 ± 0.02, *n* = 5 (LP17) in experiments with GL261 neurospheres and neutrophils or Mϕ, respectively; Results are shown as mean ± SD; the statistically significant differences are marked with asterisks (*P* < .05, Student *t-*test). (C) The graphs represent normalized transcript expression in five PDX neurosphere cell lines after treatment with SRI42127 compound versus control vehicle treatment. Results were obtained by using RNA-seq analysis and are shown as mean ± SD; the statistically significant differences are marked with asterisks (*P* < .05, Student *t-*test). (D) The graphs represent normalized tumor neurosphere proliferation in the presence of myeloid-derived cells at different treatment conditions: SRI42127 treatment versus control (vehicle treatment) and LP17 treatment versus control scrambled peptide treatment. The tumor-cell proliferation was determined by EGFP tumor-cell reporter signal. Results are shown as mean ± SD; the statistically significant differences are marked with asterisks (*P* < .05, Student *t-*test). The proliferation was significantly declined in the range of 14–18% and 14–48% in the presence of LP17 and SRI42127 treatments, respectively. (E) Representative western blot illustrates TREM1 expression in the U87 cell line at different treatment conditions. GAPDH is shown for equal protein loading.

Intercellular cell fusion events were evaluated (i) between adherent tumor cells expressing EGFP and established cell lines of neutrophils and Mϕ labeled with red-fluorescent dye (Figure 6A), and (ii) between tumor neurospheres expressing EGFP and established cell lines of neutrophils and Mϕ labeled with red-fluorescent dye (Figure 6B) under conditions that mimic hypoxia (CoCl2, 132 uM) in vitro. Figure 6A and B illustrate representative examples of cell fusion events. A significant reduction in the percentage of DP (EGFP^+^RFP^+^) cells in assays with adherent tumor cells and neutrophils and Mϕ was observed in the presence of SRI42127 (5 µM) compared to the control-vehicle treatment (Figure 6A). Next, we analyzed cell fusion events between tumor neurospheres and the established cell lines of neutrophils and Mϕ at several treatment conditions: (i) in the presence of SRI42127 (5 µM) versus vehicle treatment, and (ii) in the presence of LP17 peptide (35 ng/ml) versus scrambled control-peptide treatment (35 ng/ml). A significant reduction in fusion events between tumor neurospheres and neutrophils was observed in the presence of LP17-peptide and SRI42127 compared to the vehicle and control-peptide treatments, respectively, in the experiments with GL261 and PDX-XD456 tumor neurospheres (Figure 6B). Concordantly, cell fusion events between Mϕ and GL261 neurospheres were reduced significantly after treatments with LP17-peptide and SRI42127 versus control treatments; however, the reduction of cell fusion events between Mϕ and PDX-XD456 tumor neurospheres was only significant in the presence of LP17-peptide versus control-peptide treatment (Figure 6B). The significant reduction of cell fusion between myeloid-derived cells and tumor neutospheres by SRI42127 has been observed in the incubator with hypoxic gas mixture as well (Supplementary Figure 5).

We performed the RNA-seq analysis of five patient-derived PDX neurosphere cell lines of different molecular subtypes after SRI42127 treatment versus control and found a significant reduction in the expression of the chemo-attractants (such as CXCL2, CXCL3, SPP1) essential for the myeloid-derived cell directional migration (Figure 6C). Also, we confirmed a significant reduction in the expression of TREM1 ligands (such as HMGB1, ACTB, CIRPB, PGLYRP1) and several transcripts involved in the regulation of membrane fluidity and intercellular cell fusion with myeloid-derived cells (TNFAIP2, GJA1, MMP9, PTGES2, PTG4A, PTGA4B) (Figure 6C). Interestingly, all PDX neurospheres exhibited dominantly high expression levels of IL17D transcript and a very low level of IL17C transcript (about 100 fold less compared to IL17D). IL17D expression significantly declined after treatment with SRI42127 in all PDX subtypes; however, we detected a slight increase in the IL17C expression that might be responsible for the development of SRI42127 treatment resistance and the alternative activation of the cell signaling pathways of cell fusion in the PDX-XD456 neurospheres.

Proliferation of GL261-EGFP and XD456-EGFP neurospheres (based on the EGFP-reporter signal) was analyzed in the presence of Mϕ and neutrophils for 24 h at several treatment conditions: (i) in the presence of SRI42127 compound (5 µM) versus vehicle treatment, and (ii) in the presence of LP17 peptide (35 ng/ml) versus treatment with the scrambled control-peptide (35 ng/ml). Tumor-cell proliferation significantly declined for both cell lines in the presence of LP17-peptide and SRI42127 compound versus control-peptide and vehicle treatments, respectively (Figure 6D). The mini-ontology analysis of TREM1-dependent cell signaling pathways and transcripts expressed in gliomas of different grades based on data from the R2: platform (Supplementary Figure 6) confirmed a significant TREM1-influence on cell cycle progression in glioblastoma. Hypoxia, inflammatory response, the epithelial-mesenchymal transition, complement, KRAS, mTORC1, and TNFα via NF-kB cell signaling pathways are among the hallmarks of TREM1 related pathways in gliomas of different grades (Supplementary Figure 6) with emphasizing molecular functions such as overall enhancement of protein dimerization and endopeptidase inhibitor activity in low-grade gliomas and the enhancement of cytokine and chemokine production in high-grade gliomas (Supplementary Figure 7). Concordantly, we confirmed an increase in the TREM1 expression at the mimic hypoxic condition in the U87 cell line, differentiated neutrophil-like HL-60 cells, and primary PMN with a significant reduction of the TREM1 protein level after treatment with SRI42127 (Fig. 6E and Supplementary Figure 8).

Therefore, our results confirm the reduction of the HuR- and TREM1-dependent axis of cell fusion between glioblastoma neurospheres and myeloid-derived cells (neutrophils and Mϕ) by the inhibitor of HuR dimerization SRI42127.

## Discussion

Although glioblastoma heterogeneity has been acknowledged for nearly a decade, treatments preventing the development of glioblastoma heterogeneity and drug resistance have yet to be developed. There are several reasons for this shortage: (i) the mechanisms underlying glioblastoma heterogeneity are poorly investigated and diverse; (ii) for a long time, the focus of the investigation was placed on the glioblastoma cells alone without accounting role of the pro-inflammatory glioblastoma microenvironment. This manuscript outlines a novel axis directly involved in cell fusion events and promulgating glioblastoma progression, the TREM1^+^-HuR-dependent myeloid-derived glioblastoma microenvironment. Our work presents novel pharmacological opportunities for inhibition of the TREM1^+^-myeloid-derived glioblastoma microenvironment with a recently discovered inhibitor of HuR dimerization SRI42127.

This effort provides a detailed characterization of the myeloid-derived TREM1^+^-glioblastoma microenvironment. In agreement with recent scientific reports, we confirmed that TREM1 is highly expressed in glioblastomas of all molecular subtypes. TREM1 expression is associated with tumor-infiltrated myeloid-derived immune cells, particularly with PMN, Mϕ, and at a lower degree, with microglia.^2,8,34–36^ Our analysis revealed that TREM1 expression is a valuable biomarker of glioblastoma progression and, according to the mini-ontology analysis, positively correlates with immunosuppressive, pro-inflammatory, and angiogenic tumor phenotype in a grade-dependent manner. We confirmed a supportive role of the TREM1^+^-myeloid-derived microenvironment in cell cycle progression in glioma and an increase in the proliferation rate of the glioblastoma stem cells by tumor-infiltrated neutrophils through neutrophil extracellular traps (NETs) enriched with TREM1.^37–39^ Clinical data suggest that patients with high preoperative neutrophil to lymphocyte ratio (NLR) exhibited a poor survival rate compared to patients with low NLR.^18–20,39^ It has recently been shown that neutrophils may escort circulating tumor cells to enable cell cycle progression and metastasis.^40^ In glioma preclinical models, neutrophils promote tumor invasion, proliferation, and resistance to anti-angiogenic therapies.^41,42^ However, it is important to mention that neutrophils might also serve an anti-tumorigenic role; the necrosis promoted by neutrophil-induced ferroptosis was reported in some glioblastoma cases associated with glutathione peroxidase-4 overexpression.^43^ The TREM1^+^-positive subset of Mϕ was mostly represented by M2 polarized Mϕ, which promote migration and vascular mimicry in glioblastomas in vitro.^8^ In agreement with our results, Mϕ might contribute to glioblastoma proliferation in a TREM1-dependent manner by secreting colony stimulation factor-1 (CSF1) and stimulating the immune-suppressive microenvironment.^6,8,44,45^

In this report, we demonstrate a positive correlation between cell fusion events in glioblastoma and the TREM1^+^-myeloid-derived microenvironment. Spatial transcriptomic analysis of glioblastoma of different molecular subtypes confirmed an accumulation of TREM1 and other myeloid-cell markers in the peri-necrotic zones enriched with highly mutagenic tumor cells. Historically, the population of viable tumor cells selected from the peri-necrotic zones is hypoxia resistant, treatment-resistant, most invasive, and is enriched with de novo genotypes.^46,47^ The population of tumor cells lacking the ability for a stress-response and therefore incapable of intercellular cell fusion, tunneling nanotube (TNT) formation, and intercellular gene-transfer undergo cell death with the release of DAMP molecules. These, in combination with CXCL2, CXCL3, SPP1 cytokines, serve the role of chemo-attractants for generation of the myeloid-derived microenvironment in the peri-necrotic zones. According to several manuscripts, TREM1-ligand-dependent multimerization and activation are essential for directional migration of circulating myeloid-derived cells, production of the pro-inflammatory cytokines and fusogenic proteins.^2,9,24,48^ We found a significant reduction in the expression of HMGB1, ACTB, CIRPB, and PGLYRP1 transcripts, encoding the major TREM1 ligands, after glioblastoma-neurosphere treatment with SRI42127. Moreover, the transcriptional levels of CXCL2, CXCL3, SPP1 cytokines were also significantly reduced, in agreement with previous observations.^27^ A map of tumor-host interactions in glioma at the single-cell resolution analysis predicted several ligand-receptor cross-talks between myeloid-derived and glioblastoma cells^49^; our results suggest that the TREM1- and HuR-dependent cell-signaling pathways are the main orchestrators of these events.

The myeloid-cell heterogeneity in the tumor microenvironment, particularly of Mϕ and PMN cells, is defined as “not a single cell alike” and may reach up to 17 subsets in a single tumor.^50^ Myeloid-cells efficiently change their transcriptome and phenotype in response to the surrounding microenvironment to meet tissue demand and orchestrate suitable functional feedback. The diversity of myeloid-derived cells in glioblastomas is determined, on the one hand, by hypoxic, necrotic, nutrient-deprived, pro-inflammatory complex tissue milieu.^41,44–47^ On the other hand, the pro-inflammatory microenvironment has a significant influence on systemic immune response and brain myeloid-derived cell composition.^16^ Local and systemic TREM1-dependent myeloid-derived responses were reported in the form of TREM1^+^-positive monocytes, Mϕ, and neutrophils to brain-related injuries. The TREM1^+^-myeloid-derived cell fusogenicity with glioblastoma cells and pro-inflammatory ability might have a profound impact on transformed cell plasticity, proliferation, immune evasion, metastasis, and development of drug resistance. Therapeutic approaches to dissect and selectively, in a subset-specific manner, deplete or reprogram the myeloid-derived microenvironment in cancers are currently in great demand. Our work provides a new approach for the elimination of the TREM1^+^-myeloid-derived microenvironment in glioblastomas by using an inhibitor of HuR dimerization SRI42127, which suppressed TREM1 expression, activation, and TREM1-dependent directional myeloid-cell migration and fusion with glioblastoma cells.

## Supplementary Material

vdac149_suppl_Supplementary_Material

## References

[1] Gieryng A, Pszczolkowska D, Walentynowicz KA, Rajan WD, Kaminska B. Immune microenvironment of gliomas. Lab Investig. 2017;97(5):498–518. 28287634 10.1038/labinvest.2017.19

[2] Kluckova K, Kozak J, Szaboova K, et al Trem-1 and Trem-2 expression on blood monocytes could help predict survival in high-grade glioma patients. Mediat Inflamm. 2020;2020:1798147. 10.1155/2020/1798147 PMC7350089 32684831

[3] Delespaul L, Merle C, Lesluyes T, et al Fusion-mediated chromosomal instability promotes aneuploidy patterns that resemble human tumors. Oncogene. 2019;38(33):6083–6094. 31270395 10.1038/s41388-019-0859-6

[4] Klemm F, Maas RR, Bowman RL, et al Interrogation of the microenvironmental landscape in brain tumors reveals disease-specific alterations of immune cells. Cell. 2020;181(7):1643–1660.e17. 32470396 10.1016/j.cell.2020.05.007 PMC8558904

[5] Jung E, Osswald M, Ratliff M, et al Tumor cell plasticity, heterogeneity, and resistance in crucial microenvironmental niches in glioma. Nat Commun. 2021;12:1014. 10.1038/s41467-021-21117-3 PMC7881116 33579922

[6] DeCordova S, Shastri A, Tsolaki AG, et al Molecular heterogeneity and immunosuppressive microenvironment in glioblastoma. Front Immunol. 2020;11:1402. 10.3389/fimmu.2020.01402 PMC7379131 32765498

[7] Ochocka N, Segit P, Walentynowicz KA, et al Single-cell RNA sequencing reveals functional heterogeneity of glioma-associated brain macrophages. Nat Commun. 2021;12(1):1151. 10.1038/s41467-021-21407-w PMC7895824 33608526

[8] Kong Y, Feng Z-C, Zhang Y-L, et al Identification of immune-related genes contributing to the development of glioblastoma using weighted gene co-expression network analysis. Front Immunol. 2020;11:1281. 10.3389/fimmu.2020.01281 PMC7378359 32765489

[9] Tammaro A, Derive M, Gibot S, et al Trem-1 and its potential ligands in non-infectious diseases: from biology to clinical perspectives. Pharmacol Ther. 2017;177:81–95. 28245991 10.1016/j.pharmthera.2017.02.043

[10] Singh H, Rai V, Nooti SK, Agrawal DK. Novel ligands and modulators of triggering receptor expressed on myeloid cells receptor family: 2015–2020 updates. Expert Opin Ther Pat. 2021;31(6):549–561. 33507843 10.1080/13543776.2021.1883587 PMC8169545

[11] Nabors LB, Gillespie GY, Harkins L, King PH. HuR, an RNA stability factor, is expressed in malignant brain tumors and binds to adenine and uridine-rich elements within the 3′ untranslated regions of cytokine and angiogenic factor mRNAs. Cancer Res. 2001;61(5):2154–2161. 11280780

[12] Filippova N, Nabors LB. ELAVL1 role in cell fusion and tunneling membrane nanotube formations with implication to treat glioma heterogeneity. Cancers. 2020;12(10):3069. 33096700 10.3390/cancers12103069 PMC7590168

[13] Wang J, Leavenworth JW, Hjelmeland AB, et al Deletion of the RNA regulator Hur in tumor-associated microglia and macrophages stimulates anti-tumor immunity and attenuates glioma growth. Glia. 2019;67(12):2424–2439. 31400163 10.1002/glia.23696 PMC7008520

[14] Chongsathidkiet P, Fecci PE. Cold-inducible RNA-binding protein (CIRBP) as a biomarker to predict recurrence of brain metastases. Neuro-Oncol. 2021;23(9):1419–1420. 34036364 10.1093/neuonc/noab122 PMC8408877

[15] Denning N-L, Aziz M, Murao A, et al Extracellular CIRP as an endogenous TREM-1 ligand to fuel inflammation in sepsis. JCI Insight. 2020;5(5):e134172. 10.1172/jci.insight.134172 PMC7141396 32027618

[16] Liu Q, Johnson EM, Lam RK, et al Peripheral TREM1 responses to brain and intestinal immunogens amplify stroke severity. Nat Immunol. 2019;20(8):1023–1034. 31263278 10.1038/s41590-019-0421-2 PMC6778967

[17] Yang C, Wen H-B, Zhao Y-H, et al Systemic inflammatory indicators as prognosticators in glioblastoma patients: a comprehensive meta-analysis. Front Neurol. 2020;11:580101. 10.3389/fneur.2020.580101 PMC7575748 33117267

[18] Le Rhun E, Oppong FB, Vanlancker M, et al Prognostic significance of therapy-induced myelosuppression in newly diagnosed glioblastoma. Neuro-Oncol. 2022;24(9):1533–1545. 10.1093/neuonc/noac070 PMC9435483 35312789

[19] Bambury RM, Teo MY, Power DG, et al The association of pre-treatment Neutrophil to lymphocyte ratio with overall survival in patients with glioblastoma multiforme. J Neurooncol. 2013;114(1):149–154. 23780645 10.1007/s11060-013-1164-9

[20] Gan Y, Zhou X, Niu X, et al Neutrophil/lymphocyte ratio is an independent prognostic factor in elderly patients with high-grade gliomas. World Neurosurg. 2019;127:e261–e267. 30898756 10.1016/j.wneu.2019.03.085

[21] Aguirre LA, Montalbán-Hernández K, Avendaño-Ortiz J, et al Tumor stem cells fuse with monocytes to form highly invasive tumor-hybrid cells. OncoImmunology. 2020;9(1):1773204. 10.1080/2162402X.2020.1773204 PMC7458638 32923132

[22] Manjunath Y, Porciani D, Mitchem JB, et al Tumor-cell–macrophage fusion cells as liquid biomarkers and tumor enhancers in cancer. Int J Mol Sci. 2020;21(5):1872. 32182935 10.3390/ijms21051872 PMC7084898

[23] Gast CE, Silk AD, Zarour L, et al Cell fusion potentiates tumor heterogeneity and reveals circulating hybrid cells that correlate with stage and survival. Sci Adv. 2018;4(9):eaat7828. 10.1126/sciadv.aat7828 PMC6135550 30214939

[24] Klesney-Tait J, Keck K, Li X, et al Transepithelial migration of neutrophils into the lung requires Trem-1. J Clin Investig. 2012;123(1):138–149. 23241959 10.1172/JCI64181 PMC3533287

[25] Campbell GR, To RK, Spector SA. Trem-1 protects HIV-1-infected macrophages from apoptosis through maintenance of mitochondrial function. mBio. 2019;10(6). 10.1128/mBio.02638-19 PMC6851287 31719184

[26] Yuan Z, Syed MA, Panchal D, et al Triggering receptor expressed on myeloid cells 1 (trem-1)-mediated bcl-2 induction prolongs macrophage survival. J Biol Chem. 2014;289(21):15118–15129. 24711453 10.1074/jbc.M113.536490 PMC4031561

[27] Chellappan R, Guha A, Si Y, et al Sri-42127, a novel small molecule inhibitor of the RNA regulator hur, potently attenuates glial activation in a model of lipopolysaccharide-induced neuroinflammation. Glia. 2021;70(1):155–172. 34533864 10.1002/glia.24094 PMC8595840

[28] de Silanes IL, Zhan M, Lal A, Yang X, Gorospe M. Identification of a target RNA motif for RNA-binding protein hur. Proc Natl Acad Sci USA. 2004;101(9):2987–2992. 14981256 10.1073/pnas.0306453101 PMC365732

[29] Guha A, Waris S, Nabors LB, et al The versatile role of Hur in glioblastoma and its potential as a therapeutic target for a multi-pronged attack. Adv Drug Deliv Rev. 2022;181:114082. 34923029 10.1016/j.addr.2021.114082 PMC8916685

[30] Filippova N, Yang X, Ananthan S, et al Hu antigen R (HuR) multimerization contributes to glioma disease progression. J Biol Chem. 2017;292(41):16999–17010. 28790173 10.1074/jbc.M117.797878 PMC5641879

[31] Filippova N, Yang X, Ananthan S, et al Targeting the HUR oncogenic role with a new class of cytoplasmic dimerization inhibitors. Cancer Res. 2021;81(8):2220–2233. 33602784 10.1158/0008-5472.CAN-20-2858 PMC8137579

[32] Dixon M, Luo L, Ghosh S, et al Remodeling of the tumor microenvironment via disrupting Blimp1^+^ effector Treg activity augments response to anti-PD-1 blockade. Mol Cancer. 2021;20:150. 34798898 10.1186/s12943-021-01450-3 PMC8605582

[33] Neftel C, Laffy J, Filbin MG, et al An integrative model of cellular states, plasticity, and genetics for glioblastoma. Cell. 2019;178(4):835–849.e21. 31327527 10.1016/j.cell.2019.06.024 PMC6703186

[34] Schmid RS, Simon JM, Vitucci M, et al Core pathway mutations induce de-differentiation of murine astrocytes into glioblastoma stem cells that are sensitive to radiation but resistant to temozolomide. Neuro-Oncol. 2016;18(7):962–973. 26826202 10.1093/neuonc/nov321 PMC4896545

[35] Rincón E, Rocha-Gregg BL, Collins SR. A map of gene expression in neutrophil-like cell lines. BMC Genom. 2018;19:573. 10.1186/s12864-018-4957-6 PMC6090850 30068296

[36] Zhang L, Xu Y, Qu X. Molecular and clinical characteristics associated with elevated trem1 and its emergence as a prognostic biomarker in gliomas. Research Square. 2021. 10.21203/rs.3.rs-958851/v1.

[37] Stafford JH, Hirai T, Deng L, et al Colony stimulating factor 1 receptor inhibition delays recurrence of glioblastoma after radiation by altering myeloid cell recruitment and polarization. Neuro-Oncol. 2015;18(6):797–806. 26538619 10.1093/neuonc/nov272 PMC4864255

[38] Zha C, Meng X, Li L, et al Neutrophil extracellular traps mediate the crosstalk between glioma progression and the tumor microenvironment via the HMGB1/rage/il-8 axis. Cancer Biol Med. 2020;17(1):154–168. 32296583 10.20892/j.issn.2095-3941.2019.0353 PMC7142852

[39] Liang J, Piao Y, Holmes L, et al Neutrophils promote the malignant glioma phenotype through S100A4. Clin Cancer Res. 2013;20(1):187–198. 24240114 10.1158/1078-0432.CCR-13-1279 PMC4422653

[40] Szczerba BM, Castro-Giner F, Vetter M, et al Neutrophils escort circulating tumour cells to enable cell cycle progression. Nature. 2019;566(7745):553–557. 30728496 10.1038/s41586-019-0915-y

[41] Massara M, Persico P, Bonavita O, et al Neutrophils in gliomas. Front Immunol. 2017;8:1349. 10.3389/fimmu.2017.01349 PMC5662581 29123517

[42] Lin Y-J, Wei K-C, Chen P-Y, Lim M, Hwang T-L. Roles of neutrophils in glioma and brain metastases. Front Immunol. 2021;12:701383. 10.3389/fimmu.2021.701383 PMC8411705 34484197

[43] Yee PP, Wei Y, Kim S-Y, et al Neutrophil-induced ferroptosis promotes tumor necrosis in glioblastoma progression. Nat Commun. 2020;11:5424. 10.1038/s41467-020-19193-y PMC7591536 33110073

[44] Ye Z, Ai X, Zhao L, et al Phenotypic plasticity of myeloid cells in glioblastoma development, progression, and therapeutics. Oncogene. 2021;40(42):6059–6070. 34556813 10.1038/s41388-021-02010-1

[45] Pinton L, Masetto E, Vettore M, et al The immune suppressive microenvironment of human gliomas depends on the accumulation of bone marrow-derived macrophages in the center of the lesion. J ImmunoTher Cancer. 2019;7:4704. 10.1186/s40425-019-0536-x PMC6391795 30813960

[46] Ishii A, Kimura T, Sadahiro H, et al Histological characterization of the tumorigenic “Peri-necrotic niche” harboring quiescent stem-like tumor cells in glioblastoma. PLoS One. 2016;11(1):e0147366. 26799577 10.1371/journal.pone.0147366 PMC4723051

[47] Boyd NH, Tran AN, Bernstock JD, et al Glioma stem cells and their roles within the hypoxic tumor microenvironment. Theranostics 2021;11(2):665–683. 33391498 10.7150/thno.41692 PMC7738846

[48] Carrasco K, Boufenzer A, Jolly L, et al Trem-1 multimerization is essential for its activation on monocytes and neutrophils. Cell Mol Immunol. 2018;16(5):460–472. 29568119 10.1038/s41423-018-0003-5 PMC6474208

[49] Caruso FP, Garofano L, D’Angelo F, et al A map of tumor–host interactions in glioma at single-cell resolution. GigaScience. 2020;9(10):giaa109. 10.1093/gigascience/giaa109 PMC7645027 33155039

[50] Kiss M, Van Gassen S, Movahedi K, Saeys Y, Laoui D. Myeloid cell heterogeneity in cancer: not a single cell alike. Cell Immunol. 2018;330:188–201. 29482836 10.1016/j.cellimm.2018.02.008

