# Erratum to: Targeting the TREM1-positive myeloid microenvironment in glioblastoma

**DOI:** 10.1093/noajnl/vdac189

**Published:** 2023-01-05

This is an erratum to: Natalia Filippova, Jeffrey M Grimes, Jianmei W Leavenworth, David Namkoong, Xiuhua Yang, Peter H King, Michael Crowley, David K Crossman, L Burt Nabors, Targeting the TREM1-positive myeloid microenvironment in glioblastoma, *Neuro-Oncology Advances*, Volume 4, Issue 1, January-December 2022, vdac149, https://doi.org/10.1093/noajnl/vdac149

In the originally published version of this manuscript, the Key Points given were incorrect. The correct Key Points for this article are:

- TREM1^+^-microenvironment is a hallmark of glioblastoma and tumor/myeloid-derived cell fusion.
- TREM1 is associated with infiltration of Mϕ and PMN cells in the peri-necrotic hypoxic zones.
- HuR dimerization inhibitor suppresses the TREM1^+^-microenvironment and tumor/host cell fusion.

This has been corrected online, and the publisher apologises for the original error.

