# Supplementary material for: Targeting the TREM1-positive myeloid microenvironment in glioblastoma
Source: Neurooncol Adv. 2022 Sep 15;4(1):vdac149. doi: 10.1093/noajnl/vdac149 (PMC9555298; doi:10.1093/noajnl/vdac149)
Supplement: vdac149_suppl_Supplementary_Material [file vdac149_suppl_supplementary_material.docx]

Supplemental material for manuscript:
Targeting the TREM1- positive myeloid microenvironment in glioblastoma.

Natalia Filippova, Jeffrey M. Grimes, Jianmei W. Leavenworth, David Namkoong, Xiuhua Yang, Peter H. King, Michael Crowley, David K. Crossman, L. Burt Nabors

*Department of Neurology, Division of Neuro-oncology, UAB, Birmingham, AL, USA (NF, DN, XY, LBN); Department of Neurosurgery, Program of Immunology, UAB, Birmingham, AL, USA (JMG, JWL); Department of Neurology, Birmingham Veterans Affairs Medical Center, Birmingham, AL, USA (PHK); Department of Genetics, Heflin Center Genomics Core, UAB, Birmingham, AL, USA (MC, DKC).*

Running title: TREM1- positive glioblastoma microenvironment.

Corresponding Authors: L. Burt Nabors, M.D., Division Neuro-oncology, Department of Neurology, University of Alabama at Birmingham, FOT 1020, 510 20th Street South, Birmingham, AL 35294, USA ([bnabors@uabmc.edu](mailto:bnabors@uabmc.edu))

1. Reagents and methods.
2. Supplemental Fig. 1. TREM1 overexpression shortens glioma patient survival in a grade-dependent manner.
3. Supplemental Fig. 2. Characteristics of TREM1- positive myeloid-derived microenvironment in control (vehicle treatment) and after SRI42127 treatment in the glioblastoma models.
4. Supplemental Fig. 3. Tumor progression in GL261 immunocompetent mouse model treated with SRI42127 versus control vehicle treatment.
5. Supplemental Fig. 4. Chemotaxis of primary myeloid-derived cells towards glioblastoma cells was attenuated by HuR and TREM1 inhibitors at the mimic hypoxic condition in vitro.
6. Supplemental Fig. 5. Cell fusion events were decreased by SRI42127 in the hypoxic condition.
7. Supplemental Fig. 6. Mini ontology analysis of pathways and gene sets positively correlated with

TREM1 expression using R2: platform.

1. Supplemental Fig. 7. Biological processes, molecular function, cellular components, and cancer

hallmarks of the gene sets positively correlated with TREM1 expression.

1. Supplemental Fig. 8. TREM1 expression is downregulated by SRI42127 at the mimic hypoxic

condition.

**Reagents and methods:**

**Reagents and antibodies**

PercollTMPlus (GE HealthCare), Collagenase/Dispase (cat # 11097113001, Sigma), DNaseI (cat# 10104159001 Sigma), ACK lysis buffer (cat # A1049201, GIBCO), BBLTM Thioglycollate Medium Brewer Modified (BD), CellBrite Orange Cytoplasmic membrane Dye (Biotium), LP-17 (LQVTDSGLYRCVIYHPP) C-terminal amidation, > 85% purity (GenScript, Piscataway, NJ); control scrambled peptide (TDSRCVIGLYHPPLQVY) C-terminal amidation, > 85% purity (GenScript, Piscataway, NJ); anti-TREM1 Rabbit pAb (Proteintech), anti-mTREM1-PE conjugated (RD), anti- GAPDH Rabbit mAb (Cell Signaling), anti-HIF-1alpha Rabbit mAb (Cell Signaling), anti-HMG-1 mouse mAb (Santa Cruz Biotechnology), anti-bActin (C4) mouse mAb (Santa Cruz Biotechnology), BV421 Goat Anti-Rabbit IgG polyclonal (BD), m-IgGk BP-HRP- conjugated (Santa Cruz Biotechnology), anti-Rabbit IgG HRP-linked Ab (Cell Signaling). Live/dead-FVD (eBioscience), CD45-BV650 (Biolegend), Siglec-F- AF647 (Biolegend), PD1-FITC (Biolegend), CD11b-BV510 (Biolegend), Ly6C-APCCY7 (Biolegend), I- A/I-E-BV605 (Biolegend), TREM1-PE (FAB1187P, R&D), Ly6G-AF700 (Biolegend), F4/80-PE-Dazzle 594 (Biolegend), PD-L1-PB/BV421 (Biolegend), CD206-BV711 (Biolegend), IDO-PerCeFluor710 (Biolegend).

**Mouse GBM models and treatments**

Tumor implantation was performed at the following coordinates (1 mm posterior and 2 mm laterally to the bregma, 3 mm deep to the dura); a small burr hole was made using a dental drill; tumor cells were injected by using a 10-ul Hamilton syringe (0.5x106 cells per mouse in 3.2 ul); the burr hole was then filled with bone wax; each mouse was monitored to ensure that the implanted cells did not flow out of scull; the data was confirmed by analyzing tumor pattern in mice in vivo, and in brain slices at the endpoint of the experiments in vitro. The SRI42127 compound was administered at a dose 15 mg/Kg, twice per day by IP injection as we described.^31^ The corresponding control-vehicle treatment was administered twice per day by IP injection as described.^31^ Mice were randomly separated into two groups prior to the treatment initiation.

**Myeloid-derived cell subsets isolation and analysis**

Tumor implantation was performed at the following coordinates (1 mm posterior and 2 mm laterally to the bregma, 3 mm deep to the dura); a small burr hole was made using a dental drill; tumor cells were injected by using a 10-ul Hamilton syringe (0.5x106 cells per mouse in 3.2 ul); the burr hole was then filled with bone wax; each mouse was monitored to ensure that the implanted cells did not flow out of scull; the data was confirmed by analyzing tumor pattern in mice in vivo, and in brain slices at the endpoint of the experiments in vitro. The SRI42127 compound was administered at a dose 15 mg/Kg, twice per day by IP injection as we described.^31^ The corresponding control-vehicle treatment was administered twice per day by IP injection as described.^31^ Mice were randomly separated into two groups prior to the treatment initiation.

**Myeloid-derived cell subsets isolation and analysis**

Mice were perfused with 10 ml of ice-cold PBS through the left ventricle prior to brain harvesting. To isolate single cells, each brain was individually mechanically disassociated into small pieces (< 2 mm) followed by agitated digestion for 1 h at 37 °C in a dissociation solution (PBS supplemented with 2% FBS, 1 mg/ml collagenase/Dispase, and 0.5 mg/ml DNase I). Digested samples were washed with DMEM with 2% FBS and passed through a 70 μm cell strainer, and then myeloid-derived cells were enriched by using density gradient centrifugation for 40 min (1400rpm, break off, room temperature) in 30% Percoll. Cell pellet was washed with DMEM with 2% FBS; the red blood cells were lysed with ACK lysis buffer; cells were washed, counted, and stained for FACS analysis. Antibody panels for flow cytometry: Live/dead- FVD, CD45-BV650, Siglec-F-AF647, PD1-FITC, CD11b-BV510, Ly6C-APCCY7, I-A/I-E-BV605, TREM1-PE, Ly6G-AF700, F4/80-PE-Dazzle 594, PD-L1-PB/BV421, CD206-BV711, IDO- PerCeFluor710. Briefly, the single-cell suspension was first stained with the fixable viability dye (1:1000) in PBS for 10 min, then cells were washed with cell sorting FACS buffer (PBS, 2%FBS), were blocked with Fc block (1:200) for 10 min, and were stained with the indicated antibody mixture for 30 min. After wash with FACS buffer, cells were acquired on a BD LSR II or FACSymphony using FACSDiva software (BD Biosciences) and analyzed using FlowJo-10.7.2 software (Treestar). Intracellular staining with anti- IDO was done after cell fixation, permeabilization, and Fc blocking.

**Myeloid-derived cell isolation for in vitro experiment**

For the in vitro experiments, the myeloid-derived cell subsets (neutrophils and M ) were isolated from the peritoneal lavage fluid as described [24]. Briefly, the intraperitoneal inoculation of 1.5 ml of 4% Brewer thioglycollate medium (BD) was used for M and neutrophil recruitment to the intraperitoneal cavity; neutrophils were collected after 16 hours of stimulation; M were collected 4 days after stimulation. Cells collected from the peritoneal lavage fluid were centrifugated for 3 min at 1200 rpm, washed, and resuspended to the appropriate cell concentration in DMEM/F12 media supplemented with 2 mM L-Glutamine (Mediatech, Manassas, VA), 100U/ml Penicillin/Streptomycin (Mediatech), 10% FBS for M and in nRPMI (1x)+GlutaMAX medium (Gibco) supplemented with 100U/ml Penicillin/Streptomycin, 10% FBS for neutrophils.

**Immunohistochemistry**

Briefly, cells were washed with PBS and fixed in 3.7% paraformaldehyde for 13 min at room temperature, 0.3% Triton X-100 in PBST Buffer (0.1% Tween 20) with 2% BSA was used for 20min at room temperature for cell permeabilization, then cells were washed three times with PBST buffer, blocked with the Universal blocking buffer for 1 hour at room temperature, stained overnight with primary anti- TREM1 antibody (Proteintech; 1:100 dilution) at 4C in PBS, washed three times with PBST buffer, stained with secondary antibody (BD Horizont; 1:1000 dilution) for 1 hour at room temperature in the dark, washed three times with PBST buffer. Images were obtained with the EVOSFI imaging system.

The preparation and immunostaining of brain tissue (fixed in 4% paraformaldehyde and sliced with a thickness of 10-20 um) were performed as described previously.^31^ The antigen retrieval was performed in Tris/EDTA Buffer at 100C. Tissue was permeabilized with 0.5% Triton X in the PBST buffer with 2% BSA for 30 min at room temperature, washed three times with PBST buffer, blocked with the Universal blocking buffer for 1 hour at room temperature, stained overnight with primary anti-TREM1 antibody (Proteintech, 1:100 dilution) at 4C in PBS, washed three times with PBST buffer, stained with secondary antibody (BD Horizont, 1:1000 dilution) for 1 hour at room temperature in the dark, washed three times with PBST buffer. Images were obtained with the EVOSFI imaging system.


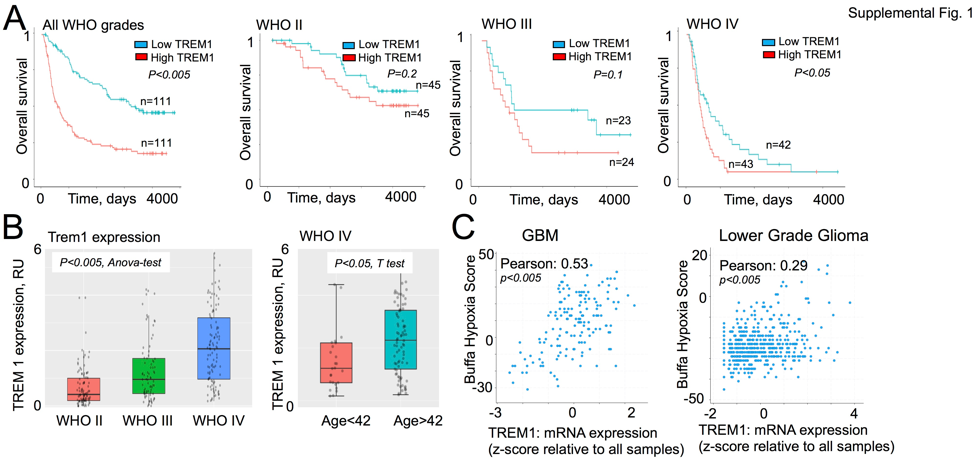


**Supplemental Fig. 1:** **TREM1 overexpression shortens glioma patient survival in a grade-dependent manner.** (**A**) Kaplan-Meier overall survival curves (x-axis represents the time since glioma diagnostic) according to the tracks “high TREM1 expression“ versus “low TREM1 expression“ (median cut-off mode) are shown for different glioma grades. The differences were significant for All WHO (P<0.005) and WHO IV (P<0.05) grade. (**B)** Graph (left) illustrates a significant increase in TREM1 levels with glioma grades. Graph (right) illustrates a significant enhancement of TREM1 levels in patients “age>42“ versus younger patients “age<42“ for WHO IV grade (P<0.05). (**C**) Plots illustrate the correlation between TREM1 expression and Buffa-Hypoxia score in grade IV gliomas (Pearson correlation 0.53, P<0.005) and lower grade gliomas (Pearson correlation 0.29, P<0.005). Data for panels A and B were obtained from Chinese Glioma Atlas; data for panel C was created using cBioPortal portal (GBM, TCGA, PanCancer Atlas and Brain Lower Grade Glioma, TCGA, PanCancer Atlas).


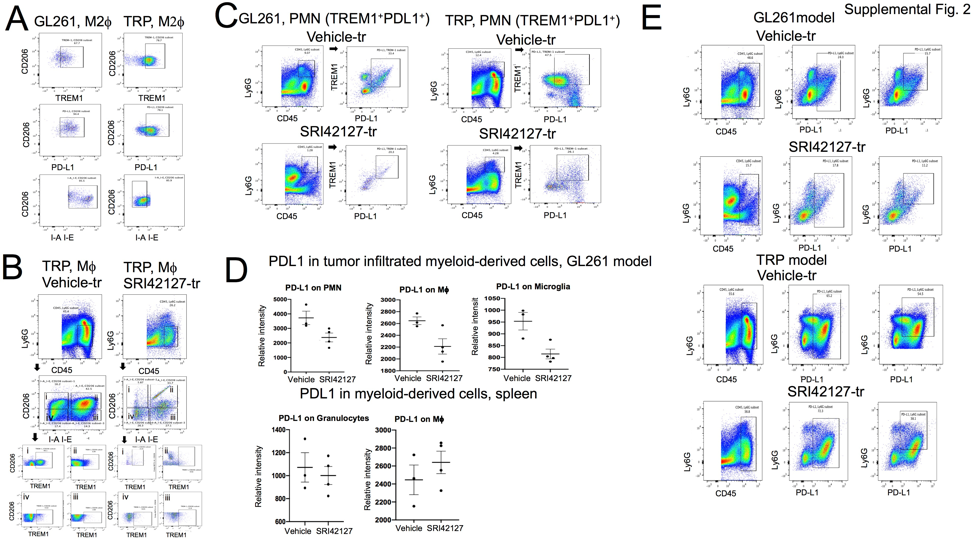


**Supplemental Fig. 2:** **Characteristics of TREM1- positive myeloid-derived microenvironment in control (vehicle treatment) and after SRI42127 treatment in the glioblastoma models.** (**A)** Plots illustrate characteristics of M2φ in GL261 and TRP glioma models. The CD45^high^CD11b^+^CD206^+^I-A/I-E^+^ population of M2φ was enriched with TREM1 in the GL261 model (up to 60%); the CD45^high^CD11b^+^CD206^+^I-A/I-E^-^ population of M2φ was enriched with TREM1 in TRP model (up to 90%). Both of these populations exhibited high PD-L1 levels. (**B)** Plots illustrate representative examples of TREM1- positive M1φ and M2φ subsets in control (vehicle treatment) versus SRI42127 treatments in TRP model. (**C)** Plots illustrate representative examples of TREM1^+^PD-L1^+^ subsets of PMN in control (vehicle treatment) versus SRI42127 treatments in GL261 and TRP models. Note a strong positive correlation between TREM1 and PDL1 expressions (R>0.5, P<0.005) in GL261 model. SRI42127 significantly diminished this population of cells from 5.5%±1% (n=5) to 2.1%±0.9% (n=5), P<0.005 of total CD45^+^CD11b^+^cells in GL261 model. (**D)** Graphs illustrate PD-L1 intensity in myeloid-derived cells purified from tumor tissue and spleens in control (vehicle treatments) versus SRI42127 treatment. The difference was significant only for PMN cells purified from tumor tissue (P<0.05). Note that resident microglia had the lowest PD-L1 intensity. **(E)** Plots illustrate representative examples of SRI4217 impact on PD-L1 in myeloid-derived subsets from GL261 and TRP models.


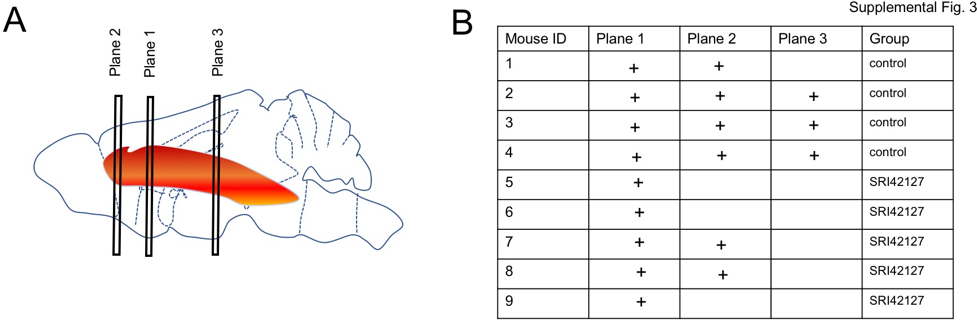


**Supplemental Fig. 3:** **Tumor progression in GL261 immunocompetent mouse model treated with SRI42127 versus control vehicle treatment.** (**A)** Schematic illustration of the mouse brain regions (plane1 corresponds to Bregma with coordinate 0 mm; plane 2 corresponds to the anterior to Bregma region with coordinates 1.7-2 mm, plane 3 corresponds to the posterior to Bregma region with coordinates 3.5-4 mm) utilized for the analysis of the tumor progression. The mouse brains were fixed in the 4% paraformaldehyde, washed in PBS, soaked in 30% sucrose, and embedded in OCT media prior to slicing as it was described.^31^ Coronal brain slices (30-50 um wide) were obtained by serial sectioning of brain tissue using cryostat as it was described.^31^ Brain slices corresponding to the brain regions (plane 1, plane 2, plane 3, two- three per region) were utilized for the staining with CellCycle 405-blue fluorescence marker (Invitrogen, Waltham, MA) for tumor cell identification. Anti-Sox2 antibody was utilized as a second marker of tumor cells using the immunohistochemistry technique. (**B**) The chart illustrates tumor progression in the mouse group treated with SRI42127 versus the control group treated with vehicles. Note that the control group exhibited the advanced tumor progression to the plane 1 and plane 3 regions (100 % and 75% of mice, respectively) versus the mouse group treated with SRI42127 (40% and 0%, respectively).


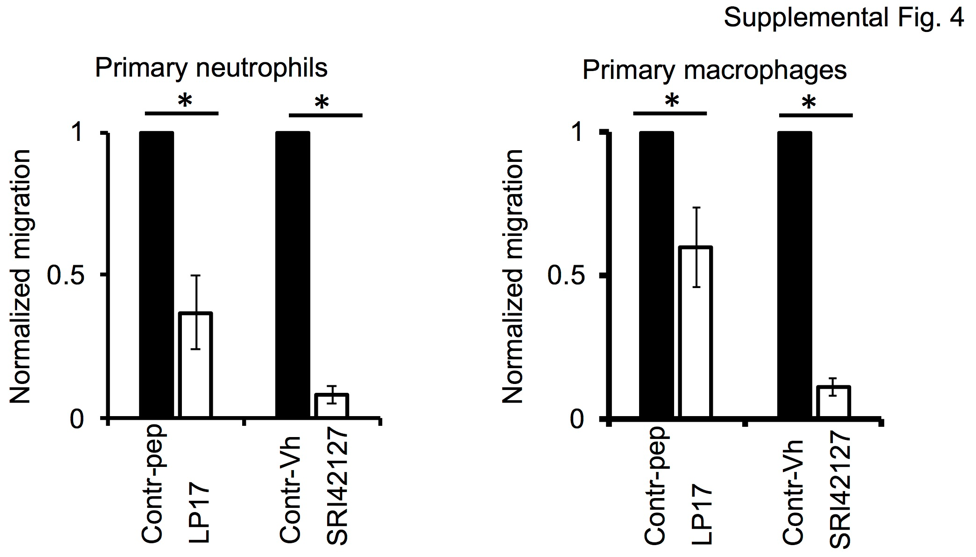


**Supplemental Fig. 4:** **Chemotaxis of primary myeloid-derived cells towards glioblastoma cells was attenuated by HuR and TREM1 inhibitors at the mimic hypoxic condition in vitro*.*** Graphs represent normalized migration of neutrophils and Mφ towards glioblastoma cells in trans-well migration assay at different treatment modalities. The normalized values of neutrophil migration towards GL261 cells detected after 12 hours of treatment were 0.37±0.13, n=4 and 0.08±0.03, n=4 in the presence of LP17 (35 ng/ml) peptide and SRI42127 (5 uM), respectively, versus control-peptide (35 ng/ml) and control vehicle treatments. The differences were significant for all conditions (p<0.05, Student *t-*test). The normalized values of Mφ migration towards GL261 cells detected after 48 hours of treatment were 0.6±0.14, n=4 and 0.11±0.03, n=4 in the presence of LP17 (35 ng/ml) peptide and SRI42127 (5 uM), respectively, versus control-peptide (35 ng/ml) and control vehicle treatments. The differences were significant for all conditions (P<0.05, Student *t*-test). Results are presented as Mean±SD.


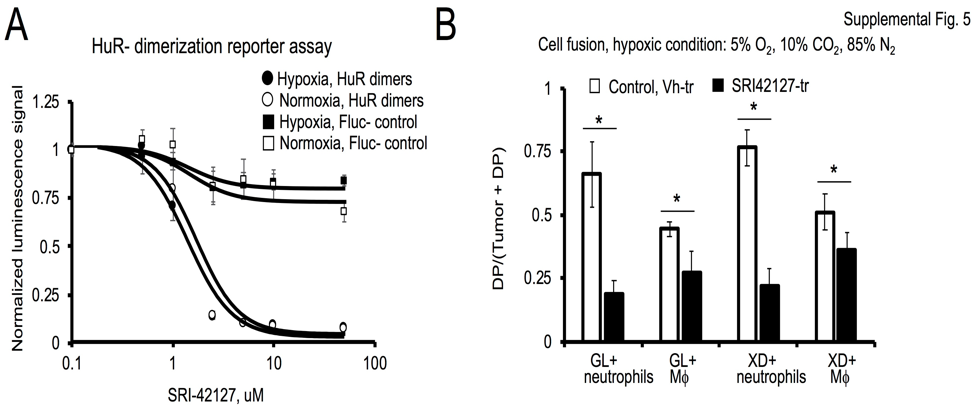


**Supplemental Fig. 5:** **Cell fusion events were decreased by SRI42127 in the hypoxic condition**. (**A**) Graph illustrates the normalized dose-response curves of inhibition of HuR- protein dimerization in the split luciferase reporter assays in normal (marked with unfilled circles) and hypoxic (marked with filled circles) conditions. The IC_50_s of SRI42127 were 1.20±0.1 uM, n=4 and 1.24±0.1 uM, n=4 in the hypoxic and normal conditions, respectively. The control Fluc signal was declined only on 16±3%, n=4 at SRI42127 concentrations up to 50 uM in the hypoxic condition. Data are presented as Mean±SD of four independent experiments. The dose-response curves were analyzed and fitted with Boltzmann equation as previously described.^31^ (**B**) The graphs represent ratios of DP (DAPI^+^Red^+^) to (DAPI^-^Red^+^ + DAPI^+^Red^+^) cells at the SRI42127 versus control (vehicle) treatment conditions. The ratios were the following: 0.66±0.12, n=5 (Vh-tr), 0.19±0.05, n=5 (SRI42127-tr), P<0.005 and 0.44±0.03, n=5 (Vh-tr), 0.27±0.08, n=5 (SRI42127-tr, P<0.005) in experiments with GL261 neurospheres and neutrophils or Mφ, respectively; 0.76±0.07, n=5 (Vh-tr), 0.21±0.07, n=5 (SRI42127-tr), P<0.005 and 0.51±0.07 n=5 (Vh-tr), 0.36±0.07, n=5, P<0.05 (SRI42127-tr) in experiments with XD456 neurospheres and neutrophils or Mφ, respectively. Cells were labeled with two different nuclear staining fluorescence dyes: i) DAPI (blue) for the myeloid-derived cells, and ii) RedDotTM1 (red) for tumor cells prior to cell fusion.


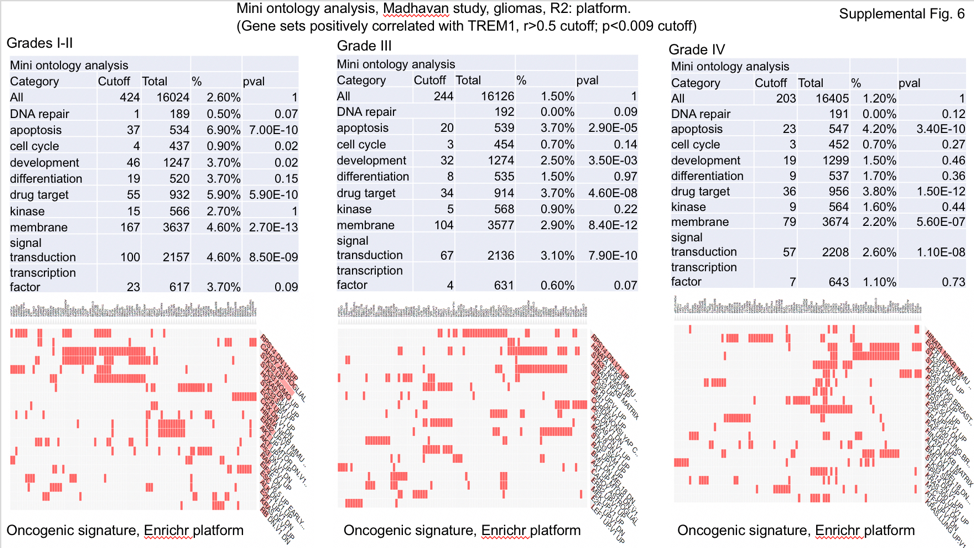


**Supplemental Fig. 6:** **Mini ontology analysis of pathways and gene sets positively correlated with TREM1 expression using R2: platform.** Madhavan study, gliomas; R>0.5 cutoffs, P<0.009 cutoffs. The plots represent the oncogenic signature of corresponding gene sets (Enrichr platform).


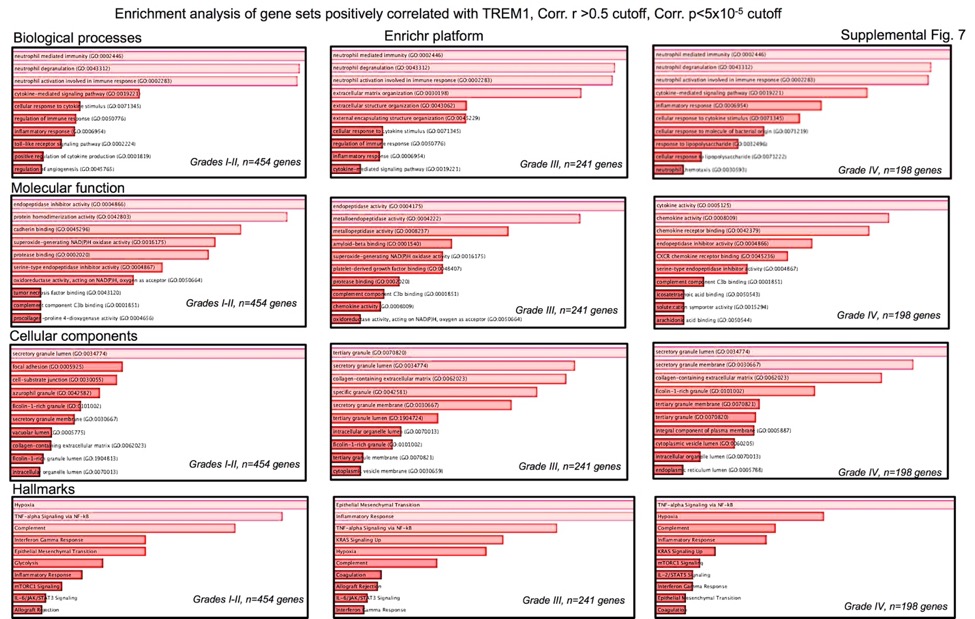


**Supplemental Fig. 7: Biological processes, molecular function, cellular components, and cancer hallmarks of the gene sets positively correlated with TREM1 expression**. Gene sets positively correlated with TREM1 were selected based on the following criteria: R>0.5 cutoffs, P<0.009 cutoffs using R2: platform, Madhavan study, gliomas, and Enrichr platform. 454 genes correspond to the above criteria from grades I-II gliomas, 241 genes correspond to the above criteria from grade III, 198 genes correspond to the above criteria from grade IV gliomas.


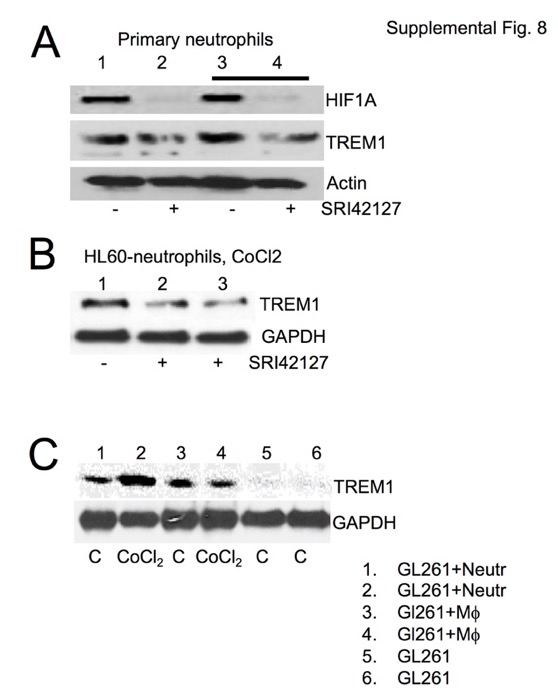


**Supplemental Fig. 8**: **TREM1 expression is downregulated by SRI42127 at the mimic hypoxic condition.** (**A)** Representative western blot illustrates downregulation of TREM1 and HIF1A expression by SRI42127 compound (5 uM, 20 hours) in primary neutrophils isolated from the peritoneal cavity and treated at the hypoxic condition (CoCl2, 132 uM). (**B)** Representative western blot illustrates downregulation of TREM1 expression by SRI42127 compound (lane 1 corresponds to control; lane 2 corresponds to SRI42127, 5 uM; lane 3 corresponds to SRI42127, 10 uM) in differentiated to neutrophil-like HL60 cells at the mimic hypoxic condition (CoCl2, 132 uM). (**C)** Representative Western blot illustrates TREM1 expression in differentiated to neutrophil-like HL60 cells and Mφ RAW267.4 cells at the control and mimic hypoxic condition (CoCl2, 132 uM) in the presence of Gl261 cells and GL261 cells alone.
